# Bridging the Gap Between Static Histology and Dynamic Organ-on-a-Chip Models

**DOI:** 10.3390/pathophysiology33010010

**Published:** 2026-01-21

**Authors:** Zheyi Wang, Keiji Naruse, Ken Takahashi

**Affiliations:** Department of Cardiovascular Physiology, Graduate School of Medicine, Dentistry and Pharmaceutical Sciences, Okayama University, 2-5-1 Shikatacho, Kita-ku, Okayama-shi 700-8558, Japan; zheyi_wang@s.okayama-u.ac.jp (Z.W.);

**Keywords:** new pathophysiology, organ-on-a-chip/OOC, dynamic disease modeling, histopathology, large-model analysis, personalized medicine

## Abstract

For more than a century, pathology has served as a cornerstone of modern medicine, relying primarily on static microscopic assessment of tissue morphology—such as H&E staining—which remains the “gold standard” for disease diagnosis. However, this conventional paradigm provides only a snapshot of disease states and often fails to capture their dynamic evolution and complex functional mechanisms. Moreover, animal models are constrained by marked interspecies differences, creating a persistent gap in translational research. To overcome these limitations, we propose the concept of New Pathophysiology, a research framework that transcends purely morphological descriptions and aims to resolve functional dynamics in real time. This approach integrates Organ-on-a-Chip (OOC) technology, multi-omics analyses, and artificial intelligence to reconstruct the entire course of disease initiation and to enable personalized medicine. In this review, we first outline the foundations and limitations of traditional pathology and animal models. We then systematically summarize more than one hundred existing OOC disease models across multiple organs—including the kidney, liver, and brain. Finally, we elaborate on how OOC technologies are reshaping the study of key pathological processes such as inflammation, metabolic dysregulation, and fibrosis by converting them into dynamic, mechanistic disease models, and we propose future perspectives in the field. This review adopts a relatively uncommon classification strategy based on pathological mechanisms (mechanism-based), rather than organ-based categorization, allowing readers to recognize shared principles underlying different diseases. Moreover, the focus of this work is not on emphasizing iteration or replacement of existing approaches, but on preserving past achievements from a historical perspective, with an emphasis on overcoming current limitations and enabling new advances.

## 1. Introduction

Pathology, derived from the Greek pathos (suffering) and logos (study), has served as the cornerstone of modern medicine for over a century. Microscopic examination of tissue morphology based on Hematoxylin and Eosin (H&E) staining has long been regarded as the “gold standard” for disease diagnosis, prognosis assessment, and treatment decision-making [1]. This traditional paradigm relies on the precise identification of structural abnormalities—ranging from cellular atypia to architectural distortion—providing an indispensable “snapshot” of the disease state. However, as medicine advances toward an era of precision and personalization, the limitations of traditional methods are becoming increasingly prominent. Histopathology inherently captures a static point in time; much like a “crime scene photo”, it reveals the consequences of disease but often obscures its dynamic evolutionary process. To understand mechanisms, researchers have long relied on animal models. Although these in vivo systems provide a systemic physiological context, significant interspecies differences exist in genetics, immunology, and metabolism [2]. Despite successes in preclinical animal studies, the high failure rate of drug candidates in clinical trials highlights a profound “translational gap”.

**Figure 1:**
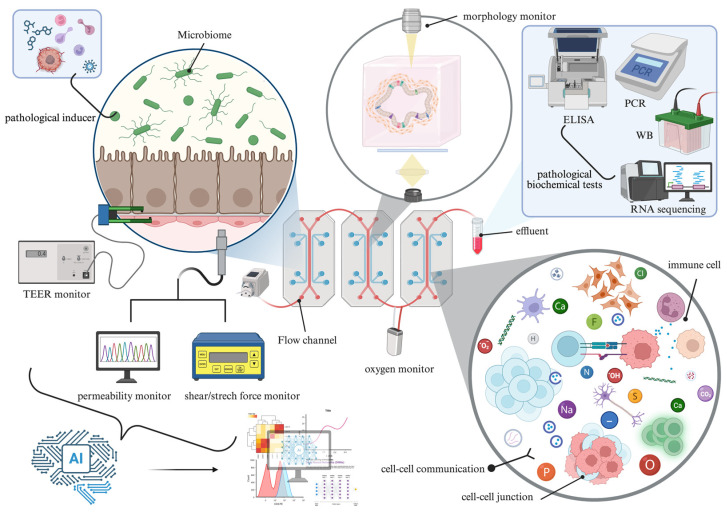
New pathophysiological research platform system. Pump-driven perfusion supports modular platforms where cells grow in predefined architectures using membranes or hydrogels, allowing real-time microscopic monitoring. Pathological stimuli (cytokines, drugs, viruses, physical factors) or patient-derived cells can be introduced. Fluorescence assays and biochemical readouts track intercellular communication, while integrated sensors measure TEER, flow rate, shear stress, pressure, permeability, and oxygen levels, with effluent analysis enabling dynamic molecular profiling. The arrows indicate the direction of the AI-based data processing workflow. Created in BioRender. WANG, Z. (2025) https://BioRender.com/zpy6p1m, accessed on 14 December 2025.

In recent years, the convergence of technologies driven by microfluidic engineering [3,4,5,6], biomaterials, iPSC technology [7,8,9,10], multi-omics analysis [11,12,13], and artificial intelligence [14,15,16] has transformed disease research from traditional “morphological description” to “dynamic functional analysis”. To encapsulate this emerging research paradigm, we propose the concept of “New Pathophysiology”. (Figure 1): a discipline that transcends morphological description to dissect the functional dynamics and molecular mechanisms of disease in real time. Its core focus is no longer the passive observation of structural alterations, but the real-time dissection, controllable simulation, and predictive modeling of the entire process of disease initiation and progression. To facilitate a more precise understanding of New Pathophysiology, we define it as follows:

New Pathophysiology refers to an interdisciplinary research paradigm that employs human-derived microfluidic organ models, single-cell and spatial omics technologies, real-time imaging, and AI-based analytics to systematically reconstruct dynamic disease mechanisms, intercellular interactions, and temporal processes. Its goal is to enable real-time observation of pathological events, mechanistic validation, and individualized prediction of disease progression.

To truly achieve these objectives, a platform that combines both biological fidelity and engineering controllability is required. Among the emerging technologies, the one that best meets the research needs of New Pathophysiology is the Organ-on-a-Chip (OOC). By recapitulating human organ multicellular architecture, tissue–tissue interfaces, and physicochemical microenvironments (such as fluid shear stress and mechanical stretch, key factors we explored in [17,18], also shown live imaging of vascular endothelial cells under mechanical stimulation in [19]), OOC offers a “dynamic” alternative to static histology and a “humanized” alternative to animal models. In fact, OOCs have already provided a more human-relevant platform than animal models for drug screening [20]. Notably, the US Food and Drug Administration (FDA) recently announced its encouragement of using alternatives to animal testing, such as organ chips, for new drug development [21], signaling a strategic shift where animal testing is intended to become ‘the exception rather than the norm’ in the future [22]. All of this suggests that OOCs will also become a crucial tool in the development of New Pathophysiology. Therefore, to distinguish them from static histology, we define an OOC as a system in which one or more cell types are co-cultured within a defined environment to recapitulate the structural or functional complexity of a specific organ or tissue through intercellular communication. This system is designed with controllable objectives (e.g., morphological or expression characteristics), exhibits overall coherence, and allows real-time monitoring and intervention.

This review aims to bridge the historical context of pathology with these cutting-edge technological advancements. We first review the foundations of traditional pathology and the role of animal models, highlighting both their contributions and inherent limitations. Subsequently, we introduce the concept of “New Pathophysiology”, elaborating on how microfluidic chips are revolutionizing the study of critical pathological processes such as inflammation, metabolic disorders, and fibrosis. Finally, we provide a comprehensive summary of existing OOC disease models, offering a roadmap for researchers navigating this rapidly evolving field. This review proposes a paradigm shift from “viewing morphology” to “interrogating function”, representing not merely a technological upgrade but a fundamental evolution in diagnostic thinking. In addressing the persistently high failure rates in drug development [23,24], the article systematically elucidates the interdisciplinary integration of engineering and pathophysiology within OOC models. By doing so, we aim to provide conceptual insights to a broad range of researchers and practitioners, and to encourage teams from diverse disciplines to collaboratively contribute to the advancement of pathophysiology and to jointly define its future.

## 2. The Historical Limitations and Breakthroughs in Pathophysiology

Traditional pathology, as the cornerstone of modern medicine, has long been recognized as the “gold standard” for disease diagnosis, grading, and prognosis assessment. Particularly in the field of oncology, pathological diagnosis based on tissue morphology remains the ultimate basis for guiding Clinical Decision-making.

Constrained by the technological limitations of its time, traditional pathophysiology has focused primarily on observing the current pathological state and identifying pathological features of diseases. Nevertheless, thanks to decades of continuous mechanistic exploration within traditional pathology, we now possess a rich foundation of knowledge and a deep understanding of various disease states. Therefore, it is time to harness rapidly advancing modern technologies to further expand the boundaries of pathology and to broaden the ways in which we investigate pathological processes.

### 2.1. Traditional Pathophysiology

From a methodological perspective, traditional pathology relies primarily on histopathology examinations. Its standard workflow is a process combining physical and chemical treatments: biological samples undergo fixation, dehydration, and embedding before being sectioned into micron-level thin slices and adhered to glass slides. Subsequently, specific chemical staining methods—the most classic and universally used being Hematoxylin and Eosin (H&E) staining—are employed to enhance the contrast between the cell nucleus and cytoplasm, thereby revealing the tissue’s microstructure [25].

In this system, the acquisition of information relies entirely on Optical Microscopy. Through direct observation, pathology analyzes cellular morphological characteristics (such as atypia and the nuclear–cytoplasmic ratio), the arrangement of tissue structures (such as glandular structures and stromal reactions), and the expression of specific biomarkers. This morphological assessment, based on physical optical paths, can not only qualitatively determine the benign or malignant nature of lesions but also provide key parameters for disease Staging and Grading. The vast morphological knowledge base accumulated by traditional pathology constitutes the training foundation and verification standard for all currently emerging diagnostic technologies (including digital pathology and artificial intelligence algorithms) [26].

Precisely because of this, inter-observer variability and intra-observer variability have led to inconsistencies in the application of pathology. When researching pathological mechanisms, traditional pathology generally utilizes the direct study of the aforementioned human pathological tissues [27] or animal models as its primary means, often by inducing similar pathological manifestations in animal models or directly using animal models that exhibit similar disease characteristics [28].

Therefore, in Section 3, we will separately elaborate on the roles played by human pathological tissues and animal pathological models in traditional pathology.

### 2.2. Technological Foundations and Conceptual Shifts in New Pathophysiology

With the integration and advancement of high-throughput molecular biology, cutting-edge imaging technologies, and computational pathology, the paradigm of disease research is shifting from morphological description to an in-depth analysis of molecular mechanisms and functional dynamics. For example, in cancer-on-a-chip systems, even when four different cell types are co-cultured, advanced live-cell imaging combined with automated image analysis enables tracking of cell-level behaviors and cell–cell interactions, as well as quantitative assessment of these processes [29]. This transition marks the advent of the New Pathophysiology era. By leveraging multi-scale and multi-dimensional datasets, this field aims to uncover the underlying mechanisms of disease initiation, progression, heterogeneity, and therapeutic response.

This conceptual transition is embodied in two fundamental shifts. First, research has moved from macroscopic observation to single-cell–level resolution. Traditional pathology often treats tissues as relatively homogeneous entities [30], whereas New Pathophysiology seeks to pinpoint pathogenic cell populations and decode the intricate interaction networks among immune, stromal, and parenchymal cells [31]—at single-cell and even spatially resolved scales.

Second, the field has shifted from a static to a dynamic understanding of disease. Instead of viewing pathology as a fixed morphological state, New Pathophysiology conceptualizes disease as a continuously evolving process driven by molecular signaling dynamics [32]. Consequently, the investigative focus has transitioned from describing “what a disease looks like” to elucidating “how a disease functions and progresses”.

Another technological foundation driving this conceptual shift in New Pathophysiology is the practical implementation of AI technologies. A key reason behind this is the increasing controllability of AI, which is essential for transforming AI into a dependable productivity tool. Only AI systems that are stable, interpretable, and reproducible can meet the technological requirements of New Pathophysiology. These innovations extend across the detection of both common and rare cancers and deliver clinical-grade performance in biomarker prediction and cell-type identification. Even with limited labeled data, foundational pathology models such as Virchow enable high-impact applications [33]. As noted earlier, traditional pathology depends on human interpretation and is subject to substantial inter- and intra-observer variability—a limitation that modern AI now provides a compelling solution for.

Another limitation of traditional pathology we discussed is the absence of the time dimension. New Pathophysiology aims to reconstruct this temporal attribute: starting from a particular stage of pathological development, we can trace backward to reconstruct past pathological events [34], or infer forward to simulate possible future trajectories [35], continuously monitor and quantify disease processes and mimic spatiotemporal environmental changes in experimental systems [36], it becomes possible that in the near future we will be able to generate different branches of disease evolution. The maturation of organ-on-a-chip technology not only aligns perfectly with this temporal dimension but also introduces repeatability—allowing us to reproduce a given process multiple times until the desired information is obtained.

## 3. Classical Methods and Frontier Technologies in Pathophysiological Research

The platforms used to study pathophysiology embody our evolving expectations for research objectives. Throughout the development of traditional pathophysiology, our understanding of disease was initially grounded in the static examination of human pathological tissues and later advanced by transplanting known mechanisms into animal models for dynamic investigation. Today, with the advent of cutting-edge technologies, we are able to reconstruct new human-relevant pathophysiological models in vitro, achieving a level of fidelity that was previously unattainable.

Therefore, we will first review the classical human-tissue-based research platforms and animal model platforms in pathophysiology, and then discuss the emerging organ-on-a-chip platform.

### 3.1. Human Subject

Human pathology is the science that studies the nature of human diseases, their mechanisms of occurrence, and the structural and functional changes they cause. For example, in processes such as inflammation, tumors, and degeneration, cells and tissues exhibit a series of observable structural changes at multiple levels, all of which are important contents of pathological research [37]. This is also the main characteristic of traditional pathology in terms of structural hierarchy and microenvironmental fidelity. Common methods include traditional optical microscopy observation, immunohistochemical staining, in situ hybridization (such as Fluorescence In Situ Hybridization), polymerase chain reaction (PCR), and next-generation sequencing (NGS) [38].

Traditional pathology employs diverse methods to study human diseases. While specific research targets vary, looking at the history of human disease research, traditional pathological methods generally fall into four main categories:

#### 3.1.1. Autopsy

As the oldest and most fundamental method, autopsy determines the cause of death and provides tissue samples. For instance, Aplastic anemia was first described by Paul Ehrlich in an autopsy report in 1888 [39]. However, tissues obtained this way represent the endpoint of pathological development.

#### 3.1.2. Biopsy (Surgical Pathology)

Utilizing techniques like surgical excision and needle puncture, this is the most common clinical method. It is used for diagnosing conditions such as adenomyosis [40] and tumors, as well as studying disease stages within the living body.

#### 3.1.3. Cytopathology

This method involves staining and observing single cells, such as cultured human primary hepatocytes [41] or cells from a lesion, and is primarily used for screening (For example, pap smears are used for cervical cancer screening [42]; sputum culture is used to screen for mycobacterium tuberculosis [43] or to identify the type of infectious pathogens; and urine cytology is used to screen for lesions within the urinary tract [44]).

#### 3.1.4. Immunohistochemistry (IHC)

By localizing specific proteins, IHC facilitates the study of pathological mechanisms and drug therapies. Notable examples include the discovery of the BCR-ABL kinase fusion protein in chronic myeloid leukemia [45] and research into signaling pathways like GPCRs [46]. However, the capacity of traditional IHC is finite. Veenstra et al., for example, noted in dermatopathology that traditional IHC is limited in the number of markers it can analyze per section, restricting deep microenvironmental analysis. They highlighted that newer technologies like Imaging Mass Cytometry (IMC)—which detects ~40 proteins simultaneously while preserving spatial info—are now needed to compensate for these deficiencies in traditional IHC and flow cytometry [47].

Despite their utility, these methods share a limitation: they capture only a “snapshot” of a specific instant in the disease’s development. Autopsies miss the process entirely, and even continuous biopsies struggle to reconstruct the full picture of disease progression. Cells in cytopathology and IHC are detached from their original temporal and histological context. In summary, traditional pathology focuses on specific, static nodes in the pathological process; it cannot simulate and reconstruct the past.

### 3.2. Animal Models

Animal models have been fundamental to advancing medical knowledge and understanding disease mechanisms. However, growing concerns about animal welfare and the relevance of research outcomes have led to increased scrutiny of their use.

Animal models have been used in various medical research, especially mechanistic studies, since the 6th century BC [48]. In 1940, Vivien Thomas and Alfred Blalock developed a dog model that mimicked tetralogy of Fallot (TOF) [49]. Since then, people began consciously translating various disease models to animals.

For example, common liver diseases include steatohepatitis, liver fibrosis, liver cancer, etc., Through dietary interventions with high fat, high fructose, and high cholesterol, pathological mouse models with extensive fibrosis can be established. C57BL/6J mice fed a standard chow or Western diet (WD) received CCl_4_, a chemical commonly used to induce liver injury and fibrosis; WD/CCl_4_ accelerated liver fibrosis and hepatocellular carcinoma (HCC), with transcriptomic profiles closely resembling human NASH, a disease characterized by hepatic steatosis, inflammation, and fibrosis [50].

However, overall, the translation from animal models to humans is not always straightforward, and the success rate is difficult to predict. Ischemic heart disease has even become the leading cause of death globally each year. Therefore, its pathological mechanisms, such as atherosclerosis, have high research value and are often discussed as an important class of cardiac diseases [51]. When exploring such pathological models, mammals, including pigs, dogs, rats, mice, and rabbits, are common modeling carriers. The heart rates of dogs and pigs are relatively close to humans, while rabbit resting heart rate is much higher than that of humans, so the left ventricular coronary perfusion is also higher than that of humans. Mice are relatively difficult to measure exercise blood flow data [52,53,54,55,56]. In contrast, large animal models (such as pigs) are closer to humans in cardiac anatomy, physiology, and lipoprotein profiles, making them more suitable for applying clinical imaging and intervention techniques, particularly for evaluating early CMD-related (Coronary Microvascular Dysfunction) neurohumoral and cardiac functional abnormalities in awake states [57].

This highlights the inherent instability of animal models: we cannot fully control individual animal physiology, and their mechanisms differ from those of humans. Hence, there is a need for alternative approaches that can capture outcomes unattainable in animal models but observable in human-relevant systems.

### 3.3. Organ-on-a-Chip

OOC platforms introduce a “temporal dimension” and “dynamic circulation”. These features enable the real-time monitoring of pharmacokinetics/pharmacodynamics (PK/PD) and inflammatory cascades, capabilities [29] that remain unattainable with traditional static histopathological sections.

This capability is made possible by the structural design of OOC systems, which aim to recreate microphysiological environments outside the body [58]. Through engineering-based control of cellular organization—such as seeding lung epithelial cells in one microfluidic chamber and vascular endothelial cells in another, separated by a porous membrane to mimic the alveolar–capillary interface—OOCs can replicate key architectural features of human organs. Mechanical breathing can also be simulated by applying cyclic vacuum pressure [59]. This integration of bioengineering and cellular systems enables the construction of physiologically relevant and highly complex disease models.

#### 3.3.1. Applications of Generalizable New Pathophysiology

In addition, the provision of a practical “toolbox” for experimental design greatly facilitates entry into this field. Researchers can reference strategies for constructing specific organ models for New Pathophysiology [Table 1], such as a blood–brain barrier (BBB) platform [60] for studying ischemia–reperfusion injury or a liver model for evaluating drug-induced liver injury (DILI) [61]. Corresponding information on chip design, cell co-culture protocols, and evaluation metrics allows investigators to efficiently implement and optimize their experimental approaches. To facilitate the retrieval of how various OOC systems are constructed within the framework of New Pathophysiology—as well as the mechanisms by which they model diseases and their corresponding applications—we have compiled a table (Table S1, references [60,61,62,63,64,65,66,67,68,69,70,71,72,73,74,75,76,77,78,79,80,81,82,83,84,85,86,87,88,89,90,91,92,93,94,95,96,97,98,99,100,101,102,103,104,105,106,107,108,109,110,111,112,113,114,115,116,117,118,119,120,121,122,123,124,125,126,127,128,129,130,131,132,133,134,135,136,137,138,139,140,141,142,143,144,145,146,147,148,149,150,151,152,153,154,155,156,157,158,159,160,161,162,163,164,165] are detailed therein) that showcases the current capabilities of OOC platforms and their substantial future potential.

To enhance intercellular connections and simulate more complex physiological and pathological mechanisms, the increasing adoption of 3D printing and bioprinting technology [Table 2] in OOC systems is highly beneficial to the development of New Pathophysiology from automated, scalable biomanufacturing [166] to the development of complex bioinks [167] and diverse printing modalities capable of constructing pathology-relevant 3D microenvironments [168].

In New Pathophysiology, organ-on-a-chip systems outperform animal models partly because they utilize human-derived cells. Complex or multi-organ models require diverse cell types, and hiPSCs—capable of differentiating into multiple lineages—provide a practical and flexible source for constructing human-relevant pathological models. Conversely, the development of organ chips also provides methods for unleashing the full potential of iPSCs [169]. The source of primary cells is a controversial issue. However, iPSCs solve this problem well. Through iPSC induction, we can obtain bone cells, muscle cells, various organ cells, etc. (Table 3). Through re-induction of normal cells, we can obtain corresponding pathological cells. Through this method, we can maintain unified system conditions to assemble pathological organ models and reproduce them multiple times to obtain more convincing results [61]. Using cell lines for amplification can be applied to large-scale research, and co-culture techniques also provide support for customizing specific pathological environments and intercellular tissue communication [170].

#### 3.3.2. Applications of Personalized New Pathophysiology

To achieve the goal of personalized pathophysiology, an increasing number of studies in recent years have begun using patient-derived cells or induced pluripotent stem cells (iPSCs) to construct patient-specific OOC models [36]. A key advantage of these patient-derived models is their ability to retain the unique features of an individual’s disease—including genetic background, metabolic status, and pathological heterogeneity [171]—thereby exhibiting highly personalized differences in drug response, inflammatory behavior, fibrosis progression, and transcriptomic signatures [172].

For example, in tumor-chip research, when comparing colorectal cancer tumor-on-chip models with the corresponding patient-derived xenografts (PDX), it was found that each patient’s chip uniquely predicted the drug sensitivities of that specific patient’s tumor, while substantial variability existed across different individuals, demonstrating that patient-specific chips can effectively function as disease avatars [173]. This phenomenon is not isolated; in lung cancer, patient-derived OOC models have been shown to accurately predict clinical responses to chemotherapy or targeted therapy—an individual-specific predictive capability entirely absent in conventional cell-line-based models [174]. In addition, when patient-derived breast tumor cells are used on-chip to evaluate the efficacy and safety of CAR-T therapy, the immunotherapy response readouts differ markedly from those obtained using standard cell lines or animal models [175].

Collectively, these studies establish an important principle: patient-specific organ-on-a-chip systems not only recapitulate general disease mechanisms but also capture the true biological variability that exists between patients, thereby providing essential support for building “differentiated, customizable patient profiles” within the framework of New Pathophysiology.

## 4. Comparison of Multiscale Disease Modeling and Research Platforms

In the previous sections, we presented the development of research platforms in pathophysiology along a timeline, focusing on three main platforms: human subjects, animal models, and OOC systems. However, to more deeply compare the characteristics of these three platforms, there is no approach more intuitive than placing them within the same pathological models. Therefore, we first compare inflammation, which was the most common focus in the early development of pathology. We then consider diseases caused by organ damage resulting from chronic inflammation, such as diabetes, and finally fibrosis, which gradually leads to functional organ loss. Using these three common pathological scenarios as examples, we perform a horizontal comparison of the current status of these three platforms in studying these pathological processes.

### 4.1. Inflammation

The progression of the vast majority of human diseases is closely associated with their pathological mechanisms. Whether it is a bacterial or viral infection (such as pneumonia or hepatitis), physical or chemical injury (such as burns or trauma), or an autoimmune disease (such as rheumatoid arthritis), the essence or primary manifestation is inflammation. Inflammation is not a single destructive process, but rather a dynamic balance of injury (destruction of the body by inflammatory agents) and anti-injury (the body’s defense and repair mechanisms).

#### 4.1.1. Human Subject

Understanding inflammation is the key to understanding the dynamic development of diseases.

Routine histological observation using H&E staining remains the gold standard for assessing inflammation. Taking common hepatitis as an example, lobular inflammation—the most primary form—consists mainly of lymphocytes (CD4+ and CD8+) and Kupffer cell aggregates (microgranulomas), with occasional neutrophils (polymorphonuclear leukocytes), particularly in the vicinity of Mallory–Denk bodies. Portal inflammation is characterized primarily by CD8+ T cells and macrophages. Consequently, by microscopically distinguishing the types and distribution of immune cells such as lymphocytes and neutrophils, we can determine the type of inflammation. Furthermore, through the application of existing semi-quantitative scoring systems—specifically by quantifying the number of inflammatory foci under 20× magnification and evaluating the degree of morphological change—we can assess the severity of the inflammation [176]. Various pathological examinations, including biopsies and blood smears, rely fundamentally on this logic of microscopic histological observation. Histopathology serves as the definitive tool for diagnosing ulcerative colitis, assessing disease severity, and identifying intraepithelial neoplasia (dysplasia) or cancer [177].

In blood examinations, aside from blood smears, the analysis of inflammatory factors as biomarkers is equally representative. Acute-phase reactants, such as the erythrocyte sedimentation rate (ESR) and C-reactive protein (CRP), have traditionally been employed as inflammatory markers and as measures of the “disease index” for both infectious and non-infectious diseases. Pro-inflammatory cytokines, including Interleukin (IL)-6, IL-1, Tumor Necrosis Factor-alpha (TNF-α), and Interferon-gamma (IFN-γ), are often active during infections; therefore, monitoring these inflammatory factors is also a crucial methodology [178].

Medical imaging is also a method for studying inflammation, offering the primary advantage of non-invasive assessment, though it is rarely used in isolation as the gold standard. Conditions such as pneumonia have long relied on initial screening via X-ray and CT scans, where the overall inflammatory presentation of tissues can suggest inflammatory imaging markers by contrast medium [179] (such as Iopamidol [180] or ionic iodinated [181]). The more precise the markers, the more accurate the imaging diagnosis. In recent years, researchers have sought to utilize various molecular imaging technologies (primarily PET, SPECT, and MRI) for more specific imaging of inflammatory responses. By incorporating specific targeting agents, the uptake of nanoparticles can be modulated, potentially allowing for the regulation of uptake by specific cell populations. Generally, the targets for various nanomaterials are predominantly monocytes and macrophages, with lymphocytes and tissue imaging playing a secondary role, covering areas such as neuroinflammation and myocardial inflammation [182].

Assessment of inflammation relies on a multilayered approach—histopathology serves as the gold standard, imaging provides non-invasive support, and hematological markers act as inflammatory biomarkers. Together, these methods help to understand the dynamic progression of disease.

#### 4.1.2. Animal Models

Investigating inflammation within animal models represents another crucial facet of pathological research. This is due not only to the ubiquity of inflammation across biological diseases but also because simulating and reconstructing inflammation in animal models is of great significance for a deeper understanding of the process.

Taking Idiopathic Interstitial Pneumonias (IIPs), a well-established category in human medicine, as an example, the US team led by Carol Reinero was the first to systematically adapt this classification system for dogs and cats, closely following the 2013 human ATS/ERS guidelines. They classified canine and feline Interstitial Lung Disease (ILD) into three main groups: Idiopathic Interstitial Pneumonias, which constitute the primary focus in this context; ILDs of known cause, including those induced by drugs, inhalants, or immune-mediated diseases; and miscellaneous ILDs. The team emphasized the importance of a Multidisciplinary Team (MDT) diagnostic approach, in which clinical data, high-resolution CT (HRCT), and lung biopsy pathology are all considered indispensable. Furthermore, the pathological manifestations of inflammation observed in humans exhibit a high degree of similarity in dog and cat models, particularly in subtypes such as Nonspecific Interstitial Pneumonia (NSIP), Cryptogenic Organizing Pneumonia (COP), and Acute Interstitial Pneumonia (AIP). However, some other subtypes, such as Lymphocytic Interstitial Pneumonia (LIP), demonstrate notable differences in the intensity and distribution of inflammatory infiltration [183].

On the other hand, the reliability of research into “Specialized Pro-Resolving Mediators (SPMs)” has sparked significant controversy in the academic community. Charles Serhan (Brigham and Women’s Hospital, Harvard Medical School) discovered the first SPM—lipoxin—in 1984. Subsequently, his team and scientists worldwide identified over 20 types of SPMs, including resolvins, maresins, and protectins. SPMs are viewed as “stop signals” for inflammation: much like a crowd dispersing, inflammation requires active intervention (akin to “riot police” clearing the area). This concept upended traditional views and propelled the field of “resolution immunology”.

However, in April 2022, Frontiers in Pharmacology published a commentary authored by 18 international scientists (including lipid analysis experts Nils Helge Schebb and Valerie O’Donnell). They pointed out that detection levels of SPMs in human samples (such as serum and urine) are extremely low (approaching “noise” levels) and insufficient to exert physiological effects. Many studies only show a “correlation” between SPMs and inflammation, lacking causal evidence that they truly “resolve” inflammation into the details, we find that one of the reasons for this controversy is that while SPMs are very easily detected in animal models with concentrations 10–1000 times higher than in humans (picomolar to nanomolar levels), in human samples, most independent laboratories either cannot detect them or find them only at “noise” levels (femtomolar levels, 10^−15^ mol/L) [184].

The examples above prove that for a long time, animal experiments have indeed served as valuable allies in studying pathological mechanisms such as inflammation. Their similarities to humans have helped us make significant strides in pathological research. Although subtle differences from humans are sometimes not enough to hinder theoretical research, as our investigations deepen, we increasingly require more precise models to support more accurate mechanistic exploration—a role for which animal models ultimately cannot serve as the optimal substitute.

#### 4.1.3. Organ-on-a-Chip

Reconstructing the dynamic process of inflammation is critically important for anchoring the interpretation of pathological progression. The OOC platform provides a system that enables real-time monitoring; during the induction of inflammation, the intensity of stimulation can be precisely controlled to model inflammatory responses of different severities. Common approaches include the addition of cytokines such as TNF-α, IL-1β, and IFN-γ [75,79,93,98,117,120,150,153], as well as induction through bacteria, viruses, or patient-derived immune cells [128,129,130,131,139,141].

As we have consistently emphasized, OOC platforms offer a uniquely irreplaceable advantage in New Pathophysiology for constructing disease models.

For example, in a microfluidic “gut–liver” connected system modeling inflammatory bowel disease (IBD), short-chain fatty acids (SCFAs) are anti-inflammatory (beneficial) under healthy conditions but become pro-inflammatory (detrimental) during active inflammatory flares [32].

In animal studies, SCFAs (such as butyrate) played an immunosuppressive and inflammation-resolving role [185]. Yet other animal studies show the same SCFAs, acting through the same receptors, recruiting neutrophils and aggravating inflammation [186].

Now, the ways in which organ-on-chip systems evaluate inflammation have also become more diverse [Table 4]. Because inflammatory mediators emerge as a dynamic process, the rapid development of artificial intelligence has greatly facilitated long-term, continuous monitoring of inflammatory markers. AI-assisted computational pipelines were used to accelerate data analysis by automating high-dimensional transcriptomic clustering and quantitative phenotypic readouts, thereby enabling faster identification of inflammation-associated cellular states and metabolic responses [126,141]. Beyond accelerating conventional analytical approaches, supervised machine learning models and convolutional neural networks (CNNs) have been integrated into organ-on-a-chip systems. By extracting features and learning patterns from chip-derived time-series imaging data, cellular morphological characteristics, barrier integrity measurements, inflammation-related fluorescent signals, and functional readouts, these models enable automated classification and prediction of ischemic injury severity, pulmonary inflammatory states, and drug response phenotypes. This integration substantially accelerates pathological assessment and drug screening processes [74,102].

Representative approaches include the use of on-chip integrated electrochemical sensors for real-time IL-6/IL-8 detection, deep-learning-based automated inflammation scoring (using bright-field/phase-contrast imaging of neutrophil clustering and epithelial damage), and VE-cadherin/ACTIN immunofluorescence combined with automated quantification of junctional disruption.

In current inflammation-on-a-chip models, inflammation is induced by exogenous or endogenous stimuli, and leukocytes—such as neutrophils [161] and lymphocytes—are introduced or recruited across an endothelial layer. The dynamic flow and vascular lining of OOC systems allow real-time, high-resolution observation of key inflammatory behaviors, including leukocyte adhesion, crawling, and transendothelial migration. These functional dynamics represent a major advantage over static 2D cultures, enabling precise quantification of leukocyte extravasation and supporting the identification of therapeutic targets that modulate inflammatory responses.

Loss of epithelial or endothelial barrier integrity is a hallmark of many inflammatory diseases, including intestinal inflammation and lung injury. The most commonly used metric to evaluate barrier function is transendothelial or transepithelial electrical resistance [93] (TEER) [154], where a decrease reflects disruption of tight junctions and impaired barrier integrity [67]. In addition, the leakage of fluorescent tracers, such as FITC-dextran, across microchannels provides a quantitative measure of barrier permeability.

Lactate dehydrogenase (LDH), a cytoplasmic enzyme released into the medium upon severe membrane damage, serves as a late-stage marker of cytotoxicity [138]. Measurement of LDH release is widely used to assess irreversible tissue damage induced by inflammatory stimuli or pharmacological agents.

Cytokines and chemokines constitute the molecular communication network of inflammation, orchestrating leukocyte recruitment and amplifying inflammatory signaling. The microfluidic design of OOC platforms allows precise sampling of effluents, which can then be analyzed using ELISA [138] or multiplex assays, such as Luminex, to quantify inflammatory cytokine profiles, including IL-6, TNF-α, and IL-1β, as well as their temporal dynamics.

Organ-on-a-chip platforms can also incorporate conventional analytical methods used in classical research systems, such as RNA sequencing and other molecular profiling techniques [69].

A major methodological advantage of OOC platforms is their ability to precisely control inflammatory stimuli, including lipopolysaccharide (LPS) and cytokines. This allows the construction of standardized dose–response and time-dependent profiles, which are often difficult to achieve in animal models. The reproducibility and fine control provided by OOC systems significantly enhance the reliability and predictive value of experimental outcomes.

### 4.2. Diabetes Mellitus

In the realm of pathology, Diabetes Mellitus (DM) holds significant importance; it is not merely an isolated endocrine metabolic disorder but a fundamental and destructive pathological state capable of systematically affecting multiple organs and systems throughout the body. The assessment of diabetes necessitates both the differentiation of its types and the evaluation of the resultant organ damage.

#### 4.2.1. Human Subject

Morphological changes in the pancreas serve as the key histological basis for differentiating between Type 1 and Type 2 diabetes [187]. Type 1 Diabetes (T1DM): The core features include insulitis, absolute insulin deficiency, and the destruction of pancreatic beta-cells. In the early stages, lymphocytic infiltration (predominantly T-cells) can be morphologically observed within the islets. As the disease progresses, islet atrophy occurs, and beta-cells significantly decrease or completely disappear. Immunohistochemistry (IHC) staining for insulin reveals minimal or absent positive cells, whereas staining for glucagon (alpha-cells) and somatostatin (delta-cells) remains persistent [188]. Type 2 Diabetes (T2DM): The core feature is amyloidosis, accompanied by a blunted response of target cells to insulin (insulin resistance) and early-stage hyperinsulinemia. Morphologically, amorphous pink material deposition (hyaline change) appears within the islets; formed by the aggregation of Islet Amyloid Polypeptide (IAPP/Amylin), these deposits displace and destroy normal islet cells. The presence of amyloid is confirmed using Congo Red staining, which demonstrates characteristic apple-green birefringence under polarized light [189].

Diabetic nephropathy (DN) represents the most classic pathological manifestation of diabetes and is the most commonly assessed condition in traditional pathological biopsies. One of the earliest observable changes under light microscopy is thickening of the glomerular basement membrane (GBM), which appears as a diffuse increase in basement membrane width when stained with Periodic Acid-Schiff (PAS). Mesangial expansion is another hallmark feature, resulting from an accumulation of extracellular matrix in the mesangial region and leading to a reduction in the glomerular filtration surface. The presence of Kimmelstiel-Wilson (K-W) nodules, considered the “gold standard” pathological feature of DN, is characterized by nodular glomerulosclerosis within the mesangium. These nodules are acellular, PAS-positive, round, and laminar, representing the most pathognomonic structural change in diabetic nephropathy. In addition, hyaline arteriolosclerosis can be observed microscopically in the afferent and efferent arterioles, which exhibit wall thickening, homogenization (hyaline change), and narrowing of the lumen [190].

Furthermore, diabetes is fundamentally a disease of vascular and microvascular pathology, with traditional pathological analysis focusing on vascular changes throughout the body. One common manifestation is hyaline arteriolosclerosis, which involves the insudation of plasma proteins into the vessel wall, leading to wall thickening, narrowing of the lumen, and subsequent ischemia in downstream tissues. In addition, diabetic retinopathy, typically assessed post-mortem, is characterized by the presence of microaneurysms, hemorrhages, and neovascularization within the retinal vasculature [191].

Diabetes exhibits distinct features in terms of classification and organ damage. When examining pathological sections, different tissue structures can be observed to assess the various impacts of diabetes.

#### 4.2.2. Animal Models

As mammals, mice are frequently employed to simulate the human physiological and pathological environment. In the study of diabetic mice, Ankit X. Sharma’s team in the United States demonstrated that glucagon receptor antagonism (GRA) improves glucose metabolism and cardiac function by promoting AMP-activated protein kinase (AMPK). This indicates that diabetic mouse models are valuable for investigating the metabolic effects of GRA in Type 2 Diabetes, successfully validating that GRA not only lowers blood glucose but also ameliorates cardiac function [192]. The capacity to validate the pleiotropic effects of drugs at the level of living organs represents a distinct advantage of animal models; they allow for the in vivo verification of drug targets and physiological mechanisms, a feat unachievable by in vitro cell experiments alone.

However, Matthias von Herrath’s team in the United States, in their discussion of animal models for human Type 1 Diabetes, clearly highlighted the prevailing sense of frustration regarding the “translation” of findings from animal models to human therapies. For instance, the vast disparity between murine and human immune responses means that immunomodulatory therapies effective in non-obese diabetic (NOD) mouse models often fail to replicate in humans. Furthermore, while animal models may exhibit tendencies toward self-healing, human disease often follows an irreversible, chronic course; additionally, many animal models are induced via chemical toxins, differing significantly from the natural pathogenesis seen in humans [193]. Although the team actively analyzed approaches such as “humanization” to align animal models more closely with human pathology, fundamental structural differences remain: in rodent islets, beta-cells are located in the core with alpha-cells at the periphery, whereas human islet cells are intermingled. This implies that paracrine signaling mechanisms differ completely between species; therefore, the inherent limitations of animal models necessitate a more cautious analysis of experimental results.

#### 4.2.3. Organ-on-a-Chip

**Figure 2:**
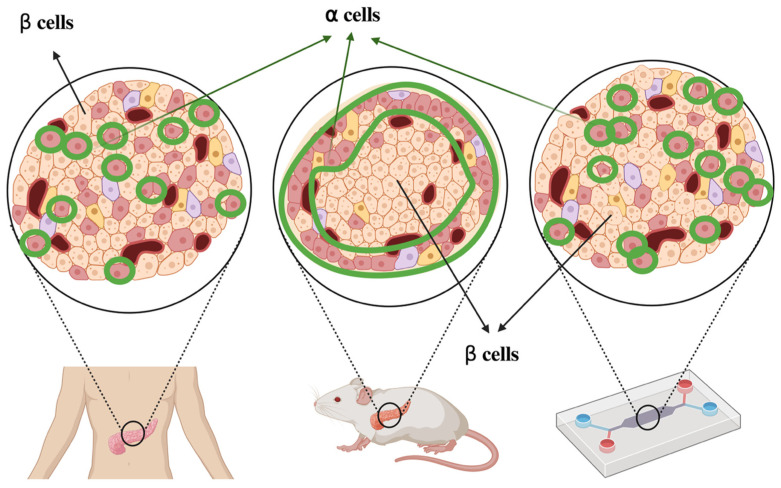
Distribution of endocrine cells in pancreatic islets across mouse, human, and islet-on-a-chip models. (The sections marked with green circles are alpha cells.) Mouse islets exhibit a clear “mantle–core” structure, with peripheral α-cells surrounding central β-cells. In contrast, human islets and the islet-on-a-chip model display a heterogeneous yet organized distribution of α- and β-cells, with cells arranged both internally and peripherally in a seemingly random pattern. Arrows denote representative cell types. Created in BioRender. WANG, Z. (2025) https://BioRender.com/e5ywtta, accessed on 14 December 2025.

Anatomical studies have shown that human pancreatic islets exhibit a patterned yet somewhat random distribution of α-cells and β-cells, whereas cells cultured directly in vitro display no such organization [194]. In contrast, mouse islets show a mantle–core architecture, with α-cells surrounding β-cells [195]. However, in an islet-on-chip system [196], mechanical pressure generated by the perfusion flow causes α-cells to distribute both internally and peripherally, reproducing the spatial organization observed in human islets (Figure 2).

The ability to recapitulate healthy human islet architecture and function also reflects the increasing technical capacity to model islet-related diseases. Through decades of research, our understanding of diabetes mechanisms has become well-established, with insulin resistance recognized as the core etiology of Type 2 Diabetes (T2D). Modeling disease directly from its causative processes—rather than relying solely on end-stage phenotypes—represents a key advantage of OOC technology within the framework of New Pathophysiology.

A major strength of OOC systems lies in their temporal fluidity. This enables reconstruction of paracrine microenvironments by co-culturing adipocytes and immune cells in physically separated yet fluidically connected compartments, where controlled microfluidics reproduce physiological shear stress and mass transport. In one representative model, human pre-adipocytes are differentiated into mature adipocytes on-chip, followed by the introduction of U937 monocytes. During obesity-induced T2D, immune cells infiltrate adipose tissue and drive chronic inflammation, ultimately causing insulin resistance. The chip’s porous barriers allow biochemical signaling between the two cell types, effectively recapitulating this pathogenic cascade [126]. It is worth emphasizing that real-time monitoring can only be achieved in OOC systems, which is not feasible in human tissue studies or animal models.

Beyond simulating the pancreatic structure itself, Organ-on-a-Chip technology possesses the functionality to combine specific organs. Culturing and differentiating human induced pluripotent stem cells (hiPSCs) allows for the generation of human liver tissue and pancreatic-like tissue (containing insulin-secreting beta-cells). In human-derived cells, C-peptide secretion is detected as an indicator of insulin production. Subsequent sequencing can identify differentially expressed genes (DEGs), revealing increased activity in transcription factors related to differentiation and maturation, such as REST, MAFB, and PBX1 [80]. However, many genes broadly expressed in human pancreatic islets show marked differences in mouse islet cells. For example, MAFB is expressed only in embryonic and neonatal β-cells in mice [197], while forced expression of REST in β-cells leads to functional impairment and glucose intolerance [198].

As emphasized earlier, diabetes is no longer considered a single disease but rather a complex endocrine system disorder. Studying the comprehensive interactions between diabetes and different organs using human tissue requires a large number of diverse samples, which may originate from different sources and thus are often difficult to compare horizontally. Animal models, on the other hand, face challenges in modeling accuracy: single-disease models are already debated, and multi-disease modeling is even more complex and difficult to validate. This is where OOC systems offer a distinct advantage—they allow customized multi-organ models with precise control over complexity [Table 5]. For example, diabetes can be modeled in combination with COVID-19 [109] or cancer [97]. Furthermore, the induction methods for diabetes in animal models are typically rapid and singular, which contradicts the decades-long natural progression of the disease in humans. In contrast, OOC systems can simulate disease progression through supraphysiological stress or acute induction [199], enabling rapid modeling while maintaining controllable complexity—an advantage unique to ex vivo platforms [200].

The real-time monitoring capabilities of Organ-on-a-Chip, combined with its ability to simulate both morphology and metabolism, allow for a comprehensive integration with pathology, unleashing unprecedented potential.

### 4.3. Fibrosis-Related Diseases

Fibrosis represents the final common pathological pathway through which almost all chronic inflammatory diseases progress toward organ failure. Consequently, it is no longer viewed merely as a symptom but as the disease entity itself—a critical “outcome” concept in pathology. When organs are subjected to prolonged, repetitive, or chronic injury—whether driven by inflammation, metabolic dysregulation, or physical trauma—an aberrant, excessive, and sustained wound-healing response triggers a pathological process of fibrosis that is typically irreversible.

#### 4.3.1. Human Subject

The investigation of fibrosis requires both the quantification of collagen deposition and the monitoring of fibroblast activation. Biopsy remains the primary method for definitive diagnosis as well as for assessing the stage and grade of fibrosis. Histological examination of H&E-stained biopsy tissues is commonly used to evaluate structural integrity and confirm the diagnosis. Quantification of fibrosis severity is typically performed using collagen-specific staining, which is considered the current “gold standard”. In the liver, for example, the METAVIR scoring system is widely applied. Common staining methods include Masson’s Trichrome, which colors collagen fibers blue, nuclei black, and cytoplasm red, and Picrosirius Red under polarized light, which allows differentiation of collagen types I and III to assess both abundance and maturity. These staining techniques are often combined with digital image analysis to calculate the proportion of fibrotic area. In addition, immunohistochemistry markers such as alpha-smooth muscle actin (alpha-SMA), Vimentin, and Desmin are employed to track the activation of myofibroblasts, providing a direct indication of fibrosis progression [201]. Furthermore, electron microscopy (EM) provides additional possibilities for ultrastructural observation.

Pulmonary fibrosis is a common fibrotic disease, with morphological examination through bronchial or lung biopsy typically revealing temporal and spatial heterogeneity, its most characteristic feature. Within a single lung lobe, this heterogeneity manifests as alternating regions of normal lung tissue, areas of active fibrosis, known as fibroblast foci, and zones of complete scarring, referred to as honeycomb change. Fibroblast foci are pale areas located at the leading edge of fibrotic regions, composed of proliferating myofibroblasts embedded within a loose extracellular matrix, representing the active centers of fibrogenesis.

Honeycomb change corresponds to end-stage cystic airspaces lined by bronchiolar epithelium and filled with mucus, with lesions predominantly located in subpleural and paraseptal regions. Clinically, pulmonary function tests are frequently employed as an adjunctive tool to assess the functional impact of these structural changes [202].

Besides pulmonary fibrosis, another common fibrotic disease occurs in the myocardium. Myocardial Interstitial Fibrosis can lead to left ventricular dysfunction and subsequent heart failure. Histomorphometry utilizing Picrosirius Red staining is the quantitative gold standard, with the Collagen Volume Fraction (CVF)—the percentage of collagen area relative to total tissue area—serving as the core metric. Furthermore, distinctions are made between specific subtypes: Replacement Fibrosis (e.g., irreversible scarring post-infarction) and Interstitial/Reactive Fibrosis (diffuse distribution, potentially reversible via pharmacotherapy in early stages) [203].

In summary, histopathological examination via tissue staining has become the universal approach for studying human pathology. However, this methodology is inherently limited by its reliance on fixed tissue sections, which offer only a static snapshot and preclude the observation of temporal dynamics.

#### 4.3.2. Animal Models

Given that fibrosis is typically an irreversible condition leading to severe consequences such as complete organ failure, investigating its potential reversibility has remained a primary objective for researchers worldwide.

The team led by Hisashi Oku in Japan utilized a bleomycin-induced murine model of pulmonary fibrosis to conduct a comparative study on the therapeutic effects of Pirfenidone versus Prednisolone. By analyzing various pulmonary cytokines at the protein level, they explored the antifibrotic efficacy and underlying mechanisms of Pirfenidone. The results demonstrated that Pirfenidone exhibited distinct antifibrotic activity in the bleomycin-induced model, whereas Prednisolone, despite its anti-inflammatory properties, failed to ameliorate fibrosis. Crucially, the mechanism of Pirfenidone extends beyond mere anti-inflammation; it involves the modulation of specific pulmonary cytokines, specifically by preserving IFN-γ levels while suppressing the production of profibrotic factors such as bFGF and TGF-β1 [204]. This study successfully elucidated the regulatory mechanisms underlying parts of the antifibrotic process, providing new strategies and insights into the potential reversibility of fibrosis.

However, an Official Workshop Report published by the American Thoracic Society (ATS) highlighted that the bleomycin mouse model can often be deceptive. The bleomycin model is typically self-limiting, with fibrosis spontaneously resolving after approximately 28 days; in stark contrast, human Idiopathic Pulmonary Fibrosis (IPF) is an irreversible, progressive, and fatal disease. Simulating a terminal condition with a “self-resolving” pathology has led to instances where therapeutic agents appear effective in animals, when in reality, they may merely be accelerating the natural recovery process. Age represents another critical factor; experiments routinely utilize young mice (8–12 weeks old), whereas human IPF is a disease of the elderly (typically over 60 years of age), and the repair mechanisms of the senescent lung differ fundamentally from those of young tissue. Furthermore, animal models are typically induced by a single insult (e.g., bleomycin or asbestos), whereas human disease is often complex and driven by multifactorial etiology [205].

In summary, while we must acknowledge the immense contribution of animal models—which, unlike static histopathology, provide complete biological dynamics and significantly enhance capabilities in drug screening and mechanistic exploration—the inherent interspecies disparities and the simplistic nature of current models constitute significant limitations in translating animal data to human clinical applications.

#### 4.3.3. Organ-on-a-Chip

In establishing fibrosis disease models, the controllability [Table 6] of OOC systems remains a unique advantage of this research platform. As mentioned earlier, NASH can lead to liver fibrosis associated with obesity, insulin resistance (diabetes), and a high-fat diet. However, to induce liver fibrosis in mice, a methionine- and choline-deficient (MCD) diet is often used, which causes rapid weight loss (cachexia) in mice and does not produce insulin resistance [206].

In contrast, organ-on-a-chip allows precise control over the conditions used to establish disease models. By using human hepatocytes on a liver chip and providing a high-fat, high-sugar culture environment that simulates an unhealthy human diet, the chip successfully reproduces the full progression from lipid accumulation to inflammation and ultimately fibrosis [207].

Organ-on-a-Chip technology, by constructing biomimetic three-dimensional microenvironments, not only recapitulates cell–matrix interactions characteristic of fibrosis but also incorporates physical factors such as fluid shear stress and mechanical strain. This capability enables the dynamic simulation and real-time monitoring of fibrotic pathological mechanisms.

Simulation Strategies for fibrosis and pathological conditions have evolved beyond simple chemical induction to “replicate” pathological environments through multidimensional approaches involving biochemical stimulation, physical mechanical forces, and 3D matrix reconstruction. The classical method involves administering TGF-beta1 (Transforming Growth Factor-beta1) to induce the transdifferentiation of fibroblasts into myofibroblasts, resulting in excessive collagen secretion. For instance, in a cardiac fibrosis model, varying degrees of fibrotic load were successfully simulated by modulating the ratio of cardiomyocytes to fibroblasts and supplementing with TGF-beta1 [105]. Furthermore, the use of biomaterials such as GelMA (methacrylated gelatin) to construct 3D hydrogel scaffolds allows for the simulation of the physical stiffness and spatial architecture of the in vivo extracellular matrix (ECM), thereby promoting physiological interactions between endothelial and supporting cells, as well as vascular network formation [71].

Fibrosis is fundamentally a physical issue as well as a chemical one, necessitating the introduction of Mechanobiology. This application is important. As mentioned earlier, bleomycin-induced fibrosis in mouse models is self-limiting and reversible, whereas organ-on-a-chip systems can maintain the model in a progressive state through continuous mechanical stimulation [208]. Research has indicated that static culture conditions alone can induce the pathological activation of cardiac fibroblasts. By applying 10% cyclic mechanical strain (1 Hz) to mimic the heartbeat, the quiescent state of fibroblasts can be maintained, and the fibrotic phenotype can even be reversed at the transcriptomic level [68]. In renal models, the introduction of fluid shear stress is critical for maintaining the polarity, microvilli formation, and transport functions of proximal tubule epithelial cells [107]. Additionally, the precise recapitulation of genetic pathologies represents another significant advantage.

Advantages over traditional methods: While traditional pathology relies primarily on histological staining, organ-on-a-chip platforms enable the measurement of organ function. For example, cardiac fibrosis models can directly quantify tissue Passive Tension (reflecting stiffness) and Active Force (contractility); these metrics provide a more accurate reflection of the disease’s impact on cardiac pumping function than collagen deposition alone [105]. Conventional culture dishes fail to provide cardiac beating or renal tubular fluid flow, leading to phenotypic drift. By simulating cyclic strain and shear stress, organ chips restore the physiological baseline of cells, rendering drug screening results more clinically relevant [68]. Furthermore, label-free real-time imaging is facilitated by the optical transparency of the chips; Second Harmonic Generation (SHG) microscopy can be used to directly observe and quantify the deposition and alignment of collagen fibers without the need for staining [105].

Physiological and biochemical monitoring also offers significant advantages. Endocytosis (Transport Function) is assessed in renal tubule models by perfusing FITC-albumin, allowing for real-time imaging to monitor the reabsorption capacity of cells and evaluate proximal tubule function [107]. YAP/TAZ Nuclear Localization serves as a key pathway indicator for mechanotransduction and fibrosis activation [68]. alpha-SMA Expression acts as a marker for myofibroblast activation. Finally, BNP (Brain Natriuretic Peptide) Secretion, detected in collected supernatant, serves as a biomarker for heart failure and stress [105].

As mentioned earlier, the development of pathology occurs in stages, and fibrosis often begins with inflammation as an early manifestation. To study the progression from inflammation to fibrosis with precision, it is necessary to fully control this process. Beyond controlling fibrosis in a single organ, such as renal fibrosis [140], regulating the disease progression itself is another unparalleled advantage of OOC systems. For example, the transition from pneumonia to pulmonary fibrosis [120], lung injury to pulmonary fibrosis [81], or osteoarthritis to bone fibrosis transforms [127] what was once a complex and highly stochastic pathological process into one that can now be precisely controlled. Unlike in animal models, where administering a drug affects the experimental subject, the animal itself is a complex, intact system with many unknown mechanisms and uncontrollable processes, making it nearly impossible to regulate the entire system using a single-factor approach. In contrast, OOC combined with New Pathophysiology can achieve this goal perfectly.

## 5. Conclusions and Perspectives

### 5.1. Summary

For over a century, traditional pathology has served as the bedrock of medical diagnosis, providing indispensable structural “snapshots” of disease states through static histology. However, the transition from morphological observation to mechanistic understanding has historically relied on animal models, which—despite their systemic complexity—are often hampered by significant interspecies discrepancies and a profound translational gap.

In this review, we proposed the concept of “New Pathophysiology” to define the paradigm shift toward dissecting the functional dynamics of human disease in real time. We identified OOC systems as the technological cornerstone of this new era. By integrating microfluidics, human-derived cells (iPSCs), and multi-omics readouts, OOCs bridge the critical gap between the static resolution of clinical histology and the dynamic, yet often unpredictable, physiology of animal models. As demonstrated through the comparative analysis of inflammation, metabolic disorders, and fibrosis, OOCs offer superior controllability and human relevance, enabling the reconstruction of temporal pathological events—from initiation to progression—that traditional methods fail to capture. As regulatory bodies like the FDA increasingly endorse alternative methods, the adoption of OOC-driven New Pathophysiology promises to revolutionize disease modeling, accelerate drug discovery, and ultimately realize the goals of precision medicine.

### 5.2. Future Perspectives

As we stand on the horizon of this new pathophysiological era, several key directions will define the future trajectory of disease research and drug discovery:

From Single-Organ to Multi-Organ [Table 7] “Body-on-a-Chip” While current OOC models excel at simulating focal pathologies (e.g., lung fibrosis or kidney tubulopathy [111]), human diseases—especially metabolic disorders like Diabetes Mellitus and systemic inflammatory syndromes—are inherently multi-systemic. Future research must prioritize the development of interconnected Multi-Organ-on-a-Chip (MOC) systems [136]. By fluidically linking liver, pancreas [80], kidney [94], and adipose tissue chips [121], researchers can model the complete PK and PD of drugs, capturing systemic effects such as inter-organ [115] metabolic signaling and off-target toxicity that single-organ models miss.

The Convergence of Biology and Artificial Intelligence: The integration of OOC with AI and computational pathology will create “Digital Twins” of biological systems. High-throughput data generated from chips—ranging from real-time TEER values [108] and metabolic readouts to live-cell imaging—far exceeds human analytical capacity. Advanced machine learning [74,126,141] will be essential to decode these multi-dimensional datasets, identifying subtle phenotypic changes and predictive biomarkers of fibrosis or inflammation long before they become morphologically visible. This synergy will automate the analysis of drug screening, reducing inter-observer variability.

By integrating iPSC technology with organ-on-a-chip systems, “Clinical Trials in a Dish” provides a powerful framework for precision medicine. By deriving organ chips from patient-specific cells, we can create “Avatars” that reflect an individual’s unique genetic background and disease susceptibility. This approach is particularly promising for rare genetic disorders (e.g., Lowe syndrome) and heterogeneous conditions like cystic fibrosis or cancer. Future clinical workflows may involve pre-screening drugs on a patient’s own “tumor-on-a-chip” or “lung-on-a-chip” to predict efficacy and toxicity, thereby minimizing risk and optimizing therapeutic strategies.

Standardization and validation for New Pathophysiology to fully complement or eventually supersede traditional animal models in regulatory frameworks, rigorous standardization is required. The field must move towards uniform biomaterials (bioinks), standardized bio-sensing integration, and reproducible fabrication protocols. Only by establishing robust, industry-standard baselines can OOC data be confidently used for regulatory submissions and widespread pharmaceutical adoption.

In summary, the future of pathology lies not in discarding the microscope, but in augmenting it with the dynamic, living microenvironments of bioengineered systems. This convergence promises to decode the “black box” of human disease mechanisms, ultimately delivering safer and more effective therapies to patients.

Today, personalized medicine and precision medicine have become central goals in humanity’s pursuit of overcoming disease and achieving better health, and OOC technologies offer a highly promising solution. By using the pathologically affected organ as the core and incorporating organs that participate in or are influenced by the disease as auxiliary components, it is possible to construct patient-specific OOC systems uniquely tailored to the individual. Such systems enable retrospective investigation of the origins of disease while simultaneously allowing prediction of therapeutic outcomes, thereby facilitating optimization of treatment strategies.

What is required is the application of now-mature technologies to faithfully replicate a patient’s pathological conditions within OOC platforms. Rapidly advancing artificial intelligence can not only accelerate the processing of information generated by these systems, but also enable high-throughput, near-real-time prediction of potential disease trajectories. Integrating AI into monitoring frameworks ensures that subtle or time-sensitive signals are not overlooked. We now possess both the capability and the ambition to deliver truly individualized precision therapies for every patient. Just as the sun will rise again the next day, a better future for pathophysiology is likewise inevitable.

## Figures and Tables

**Table 1:** Examples of pathophysiological OOC models for various organs.

Organ Type	OOC Type	Reference
Kidney	Human Proximal Tubule-on-a-Chip for Drug-Induced Kidney Injury (DIKI)	[146]
Liver	Human Liver-on-a-Chip for Hepatic Steatosis (NAFLD)	[159]
Biliary	Tubular Biliary-on-a-Chip for Biliary Injury and Repair	[79]
Brain	Human BBB-on-a-Chip for Ischemia–Reperfusion Injury	[60]
Neuro	Human Neural Tissue-on-a-Chip for Neuroinflammation	[138]
Heart	Human Cardiac Organoid-on-a-Chip for Cardiotoxicity and Myocardial Injury	[163]
Vessel	Human Aortic Smooth Muscle Cell-on-a-Chip for Thoracic Aortic Aneurysm and Dissection (TAAD)	[62]
Blood	Human Bone Marrow-on-a-Chip for Hematopoiesis and Cancer–Niche Interaction	[87]
Lymphatic Vessel	Human Lymphangion-on-a-Chip for Lymphatic Inflammation and Mechanobiology	[141]
Eye	Human Retinal Microvasculature-on-a-Chip for Barrier Dysfunction	[124]
Gum	Human Periodontal Tissue-on-a-Chip for Host–Microbe Interaction	[90]
Lung	Human Airway-on-a-Chip for Cystic Fibrosis and Host–Pathogen Interaction	[120]
Esophagus	Patient-Derived Esophageal Adenocarcinoma-on-a-Chip for Precision Oncology	[116]
Gut	Human Intestine-on-a-Chip for Enterotoxigenic *E. coli*–Induced Diarrhea	[147]
Bone	Cartilage-on-a-Chip for Biomechanically Induced Osteoarthritis	[118]
Muscle	Skeletal Muscle-on-a-Chip for Muscular Dystrophy Modeling	[91]
Skin	Inflammatory Skin-on-a-Chip for Acne-like Disease Modeling	[122]
Adipose	Isogenic White Adipose Tissue-on-a-Chip for Metabolic Inflammation and Insulin Resistance	[121]
Placenta	Human Placental Barrier-on-a-Chip for Preeclampsia Modeling	[123]
Breast	Breast Cancer Metastasis-on-a-Chip for High-Throughput Drug Screening	[114]
Uterus	Cervix-on-a-Chip for Pathological Cervical Remodeling and Preterm Birth	[149]

**Table 2:** OOC models incorporating 3D printing technology.

OOC Models	Three-Dimensional Printing Application	Reference
Alzheimer’s disease amyloid pathology-on-chip model	3D-printed miniaturized hydrogel chamber enabling controlled interstitial perfusion	[152]
Cancer extravasation-on-a-chip model for metastatic dissemination	3D-printed mold enabling open-top microfluidic architecture for 3D tumor source integration	[99]
Osteochondral arthritis-on-a-chip model for cartilage–bone inflammatory crosstalk	3D-printed biphasic construct enabling spatially separated cartilage and bone compartments	[144]
Inflammatory feto-maternal interface-on-a-chip for preterm birth modeling	3D-printed dual-chamber scaffold enabling diffusion-based feto-maternal crosstalk	[75]

**Table 3:** Methods for applying iPSCs in OOC models.

OOC Models	iPSCs Application	Reference
iPSC-derived glomerulus-on-a-chip for drug-induced nephrotoxicity	Differentiated into podocytes to reconstruct the glomerular filtration barrier	[106]
Personalized glomerulus-on-a-chip for adriamycin-induced nephrotoxicity	Differentiated into podocytes and vascular endothelial cells to reconstruct a personalized glomerular structure	[135]
Human ARPKD organoid-on-a-chip	Differentiated into kidney organoids (H9 hES and CRISPR PKHD1−/− lines) to model cystic dilatation of distal nephrons	[89]
Blood–brain barrier chip for Huntington’s disease and neuroinflammation	Differentiated into iBMECs (endothelial cells) and neural cells to recapitulate a personalized neurovascular microenvironment	[154]
Blood–brain barrier chip for ischemic stroke	Differentiated into BBB–related cells (endothelial cells forming tight junctions, pericytes) for recapitulating ischemic injury	[102]
Neuroinflammation-on-a-chip using hiPSC-derived neural tissue	Differentiated into neural system–related cells (mature neurons, astrocytes, oligodendrocytes, microglia) for inflammation	[138]
Cardiac organoid-on-a-chip for toxicity and myocardial infarction modeling	Differentiated into cardiac organoids containing cardiomyocytes, fibroblasts, and endothelial cells	[163]
Non-invasive electromechanical biosensor-on-a-chip for Duchenne muscular dystrophy cardiac modeling	Differentiated into cardiomyocytes from embryoid bodies to mimic the cardiac environment of DMD patients for studying electromechanical dysfunction and drug testing	[70]
Human cardiac fibrosis-on-a-chip	Differentiated into cardiomyocytes from hiPSCs	[105]
Angiotensin II-induced human heart-on-a-chip	Differentiated into cardiomyocytes and cardiac fibroblasts from iPSCs to mimic hypertensive heart disease and viral infection	[160]
Progressive non-genetic cardiomyopathy-on-a-chip	Differentiated into cardiomyocytes to construct 3D cardiac tissue to model non-genetic hypertrophy and fibrosis	[155]
Hereditary hemorrhagic telangiectasia (HHT1) patient-derived vessel-on-a-chip	Differentiated into vascular endothelial cells to construct 3D vascular networks to model HHT1 vascular pathology	[113]
Inflammation in a progeria-on-a-chip model	Differentiated into vascular smooth muscle cells to construct vascular structures	[129]
Stem cell-derived vessels-on-a-chip for atherosclerosis modeling	Differentiated into endothelial cells to form vascular structures and enable 3D sprouting angiogenesis	[104]
Lineage-specific vascular smooth muscle-on-a-chip for aortic aneurysm modeling	Differentiated into lineage-specific vascular smooth muscle cells (LM-SMCs, NC-SMCs, PM-SMCs) to build vascular tissue	[101]
iPSC-derived intestinal tubule-on-a-chip for IBD-like inflammation	Differentiated into multiple intestinal cell types: enterocytes, Paneth cells, neuroendocrine cells, goblet cells, and stem cells	[108]
iPSC-derived 3D skeletal muscle-on-a-chip for muscular dystrophies	Differentiated into myogenic progenitor cells (MPCs) to model DMD and limb-girdle muscular dystrophy type 2A (LGMD2A)	[91]
iPSC-derived white adipose tissue-on-a-chip for obesity and type 2 diabetes	Differentiated into adipocytes (iADIPOs) and isogenic macrophages (iMACs, M1/M2)	[121]
iPSC-derived liver-pancreas-on-a-chip for metabolic syndrome	Differentiated into hepatocyte-like cells (HLCs) and pancreatic-like tissues (PLTs, β-cell spheroids)	[80]

**Table 4:** OOC models for simulating inflammatory pathology.

Types of OOC Models	Types of Inflammation	Inducers	Reference
Tubular biliary organoid	Biliary inflammation	LPS	[79]
Blood–brain barrier chip	Neuroinflammation	TNF-α	[154]
Ischemic stroke-on-a-chip	Neuroinflammation	Ischemia/reperfusion	[102]
Neural tissue-on-chip	Neuroinflammation	TNF-α	[138]
Vessel-on-a-chip	Vascular inflammation	Viral mimic	[98]
Vascular smooth muscle chip	Vascular inflammation	Mechanical strain	[129]
Neutrophil–endothelium-on-a-chip	Sepsis-associated inflammation	Patient neutrophils/Cytomix	[161]
Lymphangion-on-a-chip	Lymphedema-associated inflammation	Pro-inflammatory cytokines	[141]
Airway-on-a-chip	CF-associated airway inflammation	Pseudomonas aeruginosa	[120]
Lung-on-a-chip	Viral/inflammatory lung response	LPS, SARS-CoV-2 pseudovirus/spike protein	[74]
Lung-on-a-chip	Acute lung/ARDS inflammation	fMLP	[153]
Respiratory mucosa-on-a-chip	Air pollution–induced inflammation	Urban Particulate Matter (UPM), TNF-α	[69]
Intestinal tubules-on-a-chip	IBD-like intestinal inflammation	Cytokine cocktail (TNF-α, IL-1β, IFN-γ)	[108]
Osteochondral-on-a-chip	Arthritis/joint inflammation	Cytokines (IL-1β, IL-6, TNF-α) and macrophage conditioned media (MCM)	[144]
Synovial tissue-on-a-chip	Osteoarthritis/synovial inflammation	TNF-α + IL-1β	[127]
Cartilage-on-a-chip	Osteoarthritis/cartilage inflammation	Hyperphysiological compression (HPC, 30% strain, 1 Hz)	[117]
Enthesis-on-a-chip	Acute and chronic enthesitis	IL-17, IL-23, TNF-α (3 days for acute; 21 days for chronic)	[86]
Adipose tissue-on-a-chip	Chronic adipose inflammation/insulin resistance	M1 macrophages (iMACs)	[121]
Feto-maternal interface-on-a-chip	Preterm birth–associated inflammation	LPS/Poly (I:C)	[75]
Lung-liver interaction-on-a-chip	Infection-induced inflammation	Inactivated NTHi and PAO1	[128]
feto-maternal interface-on-a-chip	Preterm birth–related inflammation	LPS (on maternal side)	[137]
feto-maternal interface-on-a-chip	Preterm birth–related inflammation	Cadmium (Cd)	[96]

**Table 5:** Complex OOC model combined with diabetes.

OOC Type	Interconnection	Reference
Patient-derived liver-on-a-chip for type 2 diabetes inflammation	Type 2 diabetes enhances the inflammatory response to COVID-19	[109]
Tumor-vessel-on-a-chip for cancer metastasis and diabetes-related stress	Diabetes-related dicarbonyl stress enhances tumor intravasation	[97]
Patient-derived liver- and pancreas-on-a-chip for metabolic syndrome	Diabetes/metabolic syndrome induces inflammatory responses and organ crosstalk	[80]
Adipose tissue- and immune-on-a-chip for type 2 diabetes inflammation	Type 2 diabetes enhances inflammatory responses via insulin resistance	[126]

**Table 6:** OOC for customized fibrosis modeling.

Types of Fibrosis	Inducer	Evaluation	Reference
Cardiac fibrosis	Cyclic biaxial strain, 10%, 1Hz	Transcriptomics, Mechanotransduction study, Monitoring responses in 3D microtissues	[68]
Cardiac fibrosis	TGF-β	Real-time force measurement (flexible rod deflection), Transcriptomics	[105]
Non-genetic cardiomyopathy with hypertrophy and fibrosis	Angiotensin II	Fluorescent elastic wires for force sensing, Electrical stimulation, Real-time contractility monitoring, Proteomics analysis	[155]
Lung cystic Fibrosis	Immune cell perfusion and Air-Liquid Interface	Porous membrane	[120]
Idiopathic Pulmonary Fibrosis (IPF)	Cyclic mechanical stretch (10% linear strain, 0.2 Hz)	Ultrathin membrane enables real-time observation	[81]
Synovial fibrosis associated with osteoarthritis (OA)	YAP1 overexpression/activation (mechanotransduction pathway)	COL1A1, COL3A1	[127]

**Table 7:** Multi-organ integrated OOC models.

Interconnected Organs	Recapitulated Pathology	Reference
Kidney–liver	H_2_O_2_-induced acute kidney injury (AKI) and its systemic/secondary effects on the liver	[111]
Liver–pancreas	Metabolic syndrome/diabetes–associated liver–pancreas metabolic crosstalk and inflammatory signaling (TGFβ/SMAD pathway) data	[80]
Liver–breast tumor–skin	Systemic drug metabolism and therapy-induced skin toxicity during breast cancer treatment	[132]
Adipose tissue–immune system	LPA-induced inflammation leading to insulin resistance and type 2 diabetes	[126]
Tumor–lymphatic vessel	VEGF- and interstitial flow–driven lymphangiogenesis and tumor metastasis in the tumor microenvironment	[76]
Cartilage–neuron–immune	M1 macrophage–driven inflammation inducing cartilage degeneration and pathological innervation in osteoarthritis	[95]
Liver–spleen–blood–endothelium	Hepatic-to-blood-stage malaria infection, infected erythrocyte sequestration, and hyperparasitemia	[136]
Bone–vessel–nerve	IL-1β–induced inflammatory bone disease triggering neurovascular remodeling and pain-related responses	[110]
Lung–liver	Bacteria-induced pulmonary inflammation and its systemic impact on liver function	[128]
Gut–vascular/heart	LPS-induced gut barrier disruption and inflammatory signaling along the gut–heart axis	[139]
Intestine–liver–heart–lung	Systemic evaluation of anticancer efficacy and multi-organ toxicity during lung cancer treatment	[165]
Lung–intestine	SARS-CoV-2–induced lung–gut barrier disruption and coupled immune responses	[158]

## Data Availability

No new data were created or analyzed in this study.

## Supplementary Materials

The following supporting information can be downloaded at: https://www.mdpi.com/article/10.3390/pathophysiology33010010/s1, Table S1: Integration of diverse organ-on-chip platforms with New Pathophysiology was in supplementary material.
