# Supplementary material for: Bridging the Gap Between Static Histology and Dynamic Organ-on-a-Chip Models
Source: Pathophysiology. 2026 Jan 21;33(1):10. doi: 10.3390/pathophysiology33010010 (PMC12922035; doi:10.3390/pathophysiology33010010)
Supplement: Supplementary file 1 [file pathophysiology-33-00010-s001.zip › pathophysiology-4058008-supplementary.pdf]

Supplementary Materials:

Table S1. Integration of diverse organ-on-chip platforms with New Pathophysiology

Organ	Type of microfluidic chip	Type of cell	Perfusion system	Pathophysiology	Application	Reference
Kidney	Kidney and urine model	ASCs(Adult Stem Cells) derived from human/rat kidney tissue, or human urine	3D Tubuloids (Cystic Organoids); Tubular conformation on a chip; Possessing epithelial transport function	BK Virus Infection; Wilms Tumor; Cystic Fibrosis CF	Disease Modeling (Infectious / Malignant / Genetic); Personalized Medicine; Drug Efficacy Evaluation ex vivo	[1]
	Human Proximal Tubular Cells in a Micro physiological System for injury; Vitrofluid (macro-format chip) (houses Transwell)	Primary Human Renal Proximal Tubule Epithelial Cells (RPTECs)	Pump (150 µL/min); Unidirectional flow; Fluid Shear Stress (FSS) (4.2 x 10^{-4} dyne/cm²); Transwell integration	Drug-Induced Kidney Injury (DIKI) (Induced by Colistin)	Disease modeling; Drug screening (Colistin); Cilia morphology and function study	[2]
	Glomerular Injury and nephrotoxicity mediated by ultrathin membrane; Silk Fibroin (SF) (Electrospun, Ultrathin membrane ~3.5 µm); PDMS (Chip body); Laminin-511 (Functionalization)	Human iPSCs; Podocytes (from Intermediate Mesoderm); Vascular Endothelial Cells (Isogenic)	Ultrathin membrane (mimics GBM); Size-selective filtration (Inulin/Urea pass, Albumin retain); Fenestration induction; Transmembrane cross-talk	Drug-induced Nephrotoxicity; Adriamycin (ADR); Podocyte foot process effacement; Albuminuria (compromised filtration barrier); Cytoskeletal rearrangement	Developmental biology (Morphogenesis); Disease modeling (Glomerular injury); Drug screening; Urea removal (Dialysis alternative potential)	[3]
	kidney proximal tubule-on-a-chip; PDMS	Primary Human Proximal Tubular	Continuous flow / Shear stress (0.2 dyne/cm²);	Cisplatin Nephrotoxicity; Induced by	Drug transport (Glucose/Albumin), Toxicity assessment	[4]

	Epithelial Cells (PTECs)	Porous membrane, Bilayer channel, ECM-coated (Collagen IV)	100μM Cisplatin via OCT2	(Cisplatin), Transporter activity (Pgp)	
Renal Proximal Tubule on Chip Model Phenocopies Lowe Syndrome and Dent II Disease Tubulopathy; (384-well plate based); Hydrogel (Collagen I); Mimetas (OrganoPlate® 3-lane)	HK-2 (Human proximal tubule cell line); OCRL Knockout (KO) HK-2 cells	Rocker (Gravity-driven flow) 3D tubules; Perfusion; Gene-editing; Membrane-free	Lowe Syndrome / Dent II Disease (Induced by CRISPR/Cas9 KO of OCRL)	Disease modeling; Target validation (PIP5K1A); Pathogenesis study (EMT, fibrosis)	[5]
glomerular injury chip; PDMS (elastic membrane); Emulate	(Single donor-derived): hiPSC-derived Podocytes (from IM cells); hiPSC-derived vascular Endothelial Cells (viEC)	Emulate Orb (Automated perfusion, 60 μL/h); Mechanical stretch (10% at 0.4 Hz) "Personalized" (single iPSC source); Dual-channel; Porous membrane; Mechanical stretch (mimics pulsatile blood flow)	Glomerulopathy (Induced by Adriamycin)	Disease modeling (drug-induced nephrotoxicity); Personalized medicine; Drug screening	[6]
Organoid-on-a-chip model of human ARPKD; in-house (3D-printed millifluidic chip)	iPSC-derived Kidney Organoids; H9 (hES cells) and CRISPR-mutant lines (PKHD1−/−)	Peristaltic pump; 3D organoids; Fluid flow; Mechanosensing; Cyst formation	ARPKD (Autosomal recessive polycystic kidney disease); (PKHD1−/−) induced cystic dilatation of distal nephrons	Disease modeling; Unraveling pathomechanisms (RAC1, FOS); Therapeutic target discovery; Drug testing (T-5224, R-naproxen)	[7]
A virus-induced kidney disease model; PDMS (3-layer); PET	MDCK (Canine kidney	5e-4 Pa shear force applied; -layer chip (Top channel/Membrane	Virus-induced kidney disease (induced by Pseudorabies	Disease modeling; Virus-host interaction;	[8]

	(Porous membrane); Standard soft lithography	epithelial cell line)	/Bottom well); Epithelial barrier; Shear force	Virus (PrV) infection)	Pathogenesis study (electrolyte dysfunction); Drug response (Ang II)	
	Diabetic nephropathy in a glomerulus-on-a-chip microdevice; PDMS (two-layer); 3D Matrigel; Standard soft lithography	Primary glomerular microtissues (from rat); (containing: GECs, Podocytes)	Flow rate is 2.5 μl/min; Dual-layer parallel channels (capillary/collection); 3D Matrigel (mimics basement membrane); Tissue-trapping microstructures (Crescent); Perfusion	Diabetic Nephropathy (DN) (induced by high glucose)	Disease modeling (early DN); Pathogenesis study (high glucose-induced barrier permeability, ROS production)	[9]
Liver	Hepatic steatosis in liver on chip; (384-well plate based); Hydrogel (Collagen I or Matrigel); Mimetas (OrganoPlate)	HepG2 (Human liver cell line)	Rocking platform (bidirectional flow); 3D cell culture; High-throughput (384-well); PhaseGuide™; Membrane-free	Hepatic steatosis (induced by free fatty acids OA+PA); NAFLD	Disease modeling; Drug screening (Pioglitazone, Elafibranor); Efficacy and toxicity testing	[10]
	Patient-derived chip in type 2 diabetes responses inflammatory; Nortis, Inc. (HAR-V SCC-001)	Human Hepatocytes; LSEC (Liver sinusoidal endothelial cells); Macrophages; LX-2 (Stellate cell line); (LSECs & Macrophages from T2D or non-diabetic patients)	Dynamic perfusion flow rate is 15 μl/h; Single-channel; 4-cell co-culture; 3D matrix (Collagen-fibronectin)	Type 2 Diabetes (T2D) + COVID-19 (Induced by patient cells + T2D media + S-protein overexpression)	Disease modeling; Drug screening (Tocilizumab); Pathogenesis study (Cytokine storm)	[11]
	human Liver-Chip; PDMS; Emulate	Top: Primary Human Hepatocytes (PHH); Bottom: Primary	Zoë® Culture Module (automated perfusion, 30 μL/h); Dual-channel (parenchymal/vascular); Porous	Drug-Induced Liver Injury (DILI)	Predictive toxicology; Drug screening (blinded set of 27 drugs); Economic	[12]

		Human LSECs; Human Kupffer cells; Human Stellate cells	membrane (7 μm); 3D ECM sandwich culture (hepatocytes)		analysis (of model value)	
Biliary	Tubular Biliary Organoids; PDMS; Hydrogel (Collagen I + Matrigel)	Mouse biliary organoids (from C57BL/6 mouse liver); HUVECs; Mouse fibroblasts	used laser-ablated microchannels within a hydrogel scaffold; External pumping system; Hydrogel patterning (Laser etching); Tubular structure; Perfusable branching network; Accessible lumen; Co-culture	Physical injury (mimics biliary obstruction/scarring); Chemical injury (LPS-induced biliary hyperplasia/inflammation)	Morphogenesis study; Injury repair study; Pathophysiological mechanism study (LPS inflammation); Drug transport (Rh123)	[13]
Brain	iPSC-Derived Blood-Brain Barrier Chips; PDMS; Emulate	iPSC-derived iBMECs; iPSC-derived Neural cells	Peristaltic pump; Automated platform; Dual-channel (vascular/brain); Porous membrane; Laminar flow; Real-time TEER; Cell-cell contact (via pores)	Huntington's disease (HD); MCT8 deficiency; (TNF-α induced) Inflammation	Personalized medicine; Disease modeling (hereditary); Drug permeability screening; (Transporter) mechanism study	[14]
	Modeling Ischemia-Reperfusion Injury by BBB chip; Porous PET membrane (poly(ethylene terephthalate))	HBMECs (Human brain microvascular endothelial cells); Astrocytes; Pericytes; Microglia	Swing bed (dynamic culture); High-throughput plug-in system (24 units); Tetraculture; Ischemia-Reperfusion injury; High-throughput; OGD/R stimulation	Schemic Stroke; OGD (Oxygen-Glucose Deprivation) + Reperfusion; BBB disruption (VE-cadherin↓), Apoptosis (Caspase↑), Oxidative stress (ROS↑), Mitochondrial dysfunction (MMP↓), Autophagy (Autophagosome↑)	Drug screening (AZA, EDA, FAS); Mechanism study (Transcriptome/RNA-seq); Neuroprotection assessment	[15]

	In vitro models of Parkinson's Disease (PD); Assembloid-based platform; Integrated MEA	Midbrain organoids (Dopaminergic /DA neurons); Striatal organoids (GABAergic neurons)	Integrated Microelectrode Array (MEA); Inter-organoid pathways (IOPs); Real-time electrophysiological monitoring; Spiking synchronicity; Self-assembled DA pathway	Parkinson's Disease (PD); 6-OHDA (6-hydroxydopamine); Dysfunctional dopaminergic network; Degradation of dopaminergic pathways	Drug discovery; Disease mechanisms; Efficacy assessment	[16]
	In vitro models of neurovasculature to intracerebral haemorrhage; transwells Hydrogels/Biomaterials	Endothelial cells; Pericytes; Astrocytes; Microglia	Perfusion-based systems; Vascularization techniques; Fully human-based; 3D microphysiological platforms; Complex co-culture; Clinical translation relevance	Introduction of Blood Components; Vessel Rupture & Leakage; Secondary Injury	Pre-clinical research; Elucidating pathology; Recovery mechanisms; Therapeutics discovery	[17]
	Gold nanoparticles against amyloid-β peptide in an Alzheimer's disease-on-a-chip model; PDMS/Glass; Fibrin (3D scaffold); Collagen (Coating)	Astrocytes; Pericytes; Endothelial Cells (ECs)	Gravity-driven perfusion; 3D cellular architecture; BBB integrity; Rational design GNP; Peptide D3 functionalization	Alzheimer's Disease (AD); Amyloid-β peptide (Aβ); BBB dysfunction; Aβ cytotoxicity; Endothelial damage	Drug delivery (GNP nanocarriers); Toxicity testing (Aβ inhibition); Disease modeling (AD pathology); Preclinical validation	[18]
	A miniaturized hydrogel-based in vitro model for beta-amyloid precursor protein; 3D printed); Semipermeable membrane (0.4 µm);	H4-SW cells (Human neuroglioma cells expressing APP-Swedish mutation)	Syringe pump (0.75 / 0.25 µL/min); Counter-current perfusion 3D cell encapsulation Interstitial perfusion Porous membrane	Alzheimer’s Disease (AD) modeled using H4-SW cells	Disease modeling (AD); as part of the MGBA platform; drug target investigation	[19]

	Hydrogel (Collagen/HA or Collagen/PEG)		Optically accessible / Optical accessibility			
	An ischemic stroke-on-a-chip model	Human iPSC-derived cells; Endothelial cells (forming tight junctions); Pericytes	Machine learning integration (Random Forest/LASSO); Transcriptomic profiling (WGCNA); Tight junction disruption	schemic stroke (Induced by OGD); Simulating energy failure, oxidative stress, inflammation	Drug screening (validated Coumarin); Biomarker discovery; Disease modeling	[20]
Neuro	Neural Tissue-On-Chip: A Neuroinflammation Model; PDMS; Matrigel (Tissue scaffold); Collagen Type I (Coating); Finnadvance (GPTrans-F96 plate)	Differentiated from hiPSCs (Single donor): Mature Neurons ($5x10^4$); Astrocytes ($2x10^3$); Oligodendrocytes ($7.5x10^3$); Microglia ($5x10^2$); (Ratio Neuron:Glia = 5:1); hCMEC/D3 (Human cerebral microvascular endothelial cells)	Rocker platform (bidirectional flow); Static incubation initially; Isogenic neural/glial cells; BBB integration; 3D neural tissue; EV therapy testing; Neuroinflammation modeling	Neuroinflammation; TNF-α (50 ng/mL); Cytokine release (↑TNF-α, ↑IL-6, ↑IL-1β); Tissue toxicity (LDH release)	Disease modeling (Neuroinflammation); Therapeutics evaluation (BMSC-derived EVs); Cytokine profiling (ELISA); Gene expression analysis	[21]
Heart	Cardiac Organoid-on-a-Chip	iPSC-derived Cardiac Organoids (iPSC-derived COs) (containing: Cardiomyocytes, Fibroblasts, Endothelial cells)	Microfluidic system; 3D Cardiac Organoids; Mechanical stimulation	Polystyrene nanoplastic (PS-NP) induced cardiotoxicity; Myocardial infarction (MI) model	Cardiotoxicity evaluation (dynamic observation); Vulnerability study in pathological states	[22]
	Non-invasive electromechanical cell-based biosensors for 3D cardiac	hESC (CCTL14 line); hiPSC (Patient-derived,	Static bath (Tyrode solution); Manual drug administration;	Duchenne Muscular Dystrophy (DMD);	Disease modeling (DMD characterization); Drug testing (Isoproterenol,	[23]

models; MEA (Microelectrode array, Ti/ITO electrodes); AFM Cantilever (Silicon nitride); Coating (Laminin/Fibronectin); Multichannel Systems (MEA); JPK (AFM); Bruker (Cantilever)	Duchenne Muscular Dystrophy); Embryoid body-derived Cardiomyocytes	Simultaneous electromechanical recording; Non-invasive; Electro-mechanical delay (EMD) measurement; AFM + MEA integration	Loss of dystrophin; Calcium handling defects; Altered beating-force relation	Verapamil, Calcium sensitivity); cECC study	
cardiac fibroblast transcriptome in a human cardiac fibrosis-on-a-chip; PDMS (Sylgard 184, 52 μm film); Stainless steel 316 (Chip plates); Glass (Bottom for optical access); Custom-made 24-well platform	hfCF (Human fetal cardiac fibroblasts); GelMA (Gelatin methacryloyl, 10% w/v, Stiffness ~12 kPa)	Pneumatic actuation (6 pumps); Labview control; Cyclic biaxial strain (0–25% magnitude, 1 Hz); High-throughput (up to 144 samples); 3D microtissues	Cardiac fibrosis ;Cyclic strain (10%, 1Hz) induces antifibrotic effects (↓COL1a1, ↓ACTA2/αSMA, ↑MMP1)	Mechanotransduction study; Transcriptomics; Disease modeling	[24]
Human cardiac fibrosis-on-a-chip model; PMMA (or Polystyrene); PDMS (flexible rods); Hydrogel (Fibrin); Micromilling	hiPSC-CMs (iPSC-derived cardiomyocytes); Human cardiac fibroblasts	tatic well-plate culture; 3D Microtissues; Real-time force measurement (flexible rod deflection); 3D Co-culture	Cardiac Fibrosis (Induced by TGF-β)	Disease modeling (Heart failure); Drug screening (Pirfenidone, Losartan, Carvediolol); Pathogenesis study (transcriptomics)	[25]
Hydrogel-Integrated Heart-on-a-Chip Platform for Assessment of Myocardial Ischemia	H9C2 (Rat cardiomyoblast cell line)	Pressure-controlled system (Elveflow) (40 mbar); 3D cell-hydrogel encapsulation; Single-channel; Perfusion	Myocardial ischemia (induced by Hypoxia (1% O_2))	Disease modeling; Biomarker detection (LC-MS/MS)	[26]

	Markers; PDMS (Chip); Hydrogel (Alginate-Gelatin-Collagen); Photolithography and soft lithography					
	angiotensin II-induced heart-on-a-chip disease model; 3D-printed thermoplastic elastomer; Hydrogel (Collagen); multiwell format	iPSC-derived Cardiomyocytes; iPSC-derived Fibroblasts	static well-plate culture; 3D tissue (Biowire); Nanocomposite wires (for force sensing); Electrical stimulation; Real-time contractility monitoring	Hypertensive heart disease (Ang II induced); SARS-CoV-2 infection	Disease modeling (comorbidities); Viral pathogenesis study; Drug/biologics screening (EV therapy)	[27]
	Progressive non-genetic cardiomyopathy model; Polystyrene (Chip body); POMaC (Elastic wires); Hydrogel (Fibrin-collagen); Biowire II platform	hiPSC-derived Cardiomyocytes (hiPSC-CMs); Human ventricular Fibroblasts (cFB)	static well-plate culture; 3D tissue (Biowire); Fluorescent elastic wires (for force sensing); Electrical stimulation; Real-time contractility monitoring; Co-culture	Non-genetic cardiomyopathy (Hypertrophy and fibrosis induced by Ang II)	Disease modeling (progressive); Pathological mechanism study (proteomics); Pre-clinical drug evaluation (Losartan, Relaxin, Saracatinib)	[28]
Vessel	Aorta Smooth Muscle Cell Organ-On-A-Chip Model; PDMS (Chip); PDMS (Flexible membrane)	p-HASMCs (Primary human aortic smooth muscle cells) (from healthy, BAV-TAAD, and TAV-TAAD patients)	Vacuum pump (for mechanical stretch); Peristaltic pump (for media circulation); Biomechanical strain; Dynamic perfusion (circulating)	Thoracic Aortic Aneurysm and Dissection (TAAD) (Modeled using patient-derived cells + mechanical strain)	Disease modeling (BAV-TAAD, TAV-TAAD); Pathogenesis study; Therapeutic target discovery	[29]
	3D microfluidic platform for CAVD; PDMS (Channels); PET porous	Primary porcine, seeded on porous membrane;	Peristaltic pump (recirculatory flow); Syringe pump (continuous	Calcific Aortic Valve Disease (CAVD); Myofibroblast differentiation	Mechanistic study (Shear-regulated paracrine interactions);	[30]

membrane (1 μm pores); Gel-MA (Gelatin methacrylate hydrogel, 5-15% w/v); Standard soft lithography & squeeze fabrication	Primary porcine, encapsulated in Gel-MA hydrogel	perfusion); Shear stress (20 dyn/cm²); Bilayer membrane device; 3D Heterotypic co-culture; Tunable hydrogel stiffness (2-30 kPa); Paracrine regulation; Compartmentalization	(α-SMA+); Fibrosis; Shear stress enhances VEC suppression of VIC pathological differentiation	Drug screening (Potential); Valvular biology	
Aortic valve tissue culture maintenance in microphysiological system; Polycarbonate (TIC shells/pump chip); TPU (membrane/ring)	Valvular Interstitial Cells; Valvular Endothelial Cells	Pulsatile flow; High flow (77.4 μL/s, 30 bpm); Low flow (13.4 μL/s, 150 bpm); Long-term culture (14-26 days); Bimodal viability assay; ECM maintenance; Intermediate scale tissue	Early CAVD (Calcific Aortic Valve Disease); Tissue contraction/shrinkage; Collagen fiber increase; Mass reduction	CAVD pathophysiology; Tissue culture validation; Biomechanical simulation	[31]
Low levels of physiological interstitial flow model; PDMS; Fibrin gel (ECM)	ECFC-ECs (Endothelial colony forming cell-derived endothelial cells); NHLFs (Normal human lung fibroblasts)	Hydrostatic pressure gradient; Interstitial flow (0.1–43 μm/s;3D Angiogenesis; Morphogen gradient control; Interstitial flow; Integrin mediation (alpha\beta); Gradient elimination	Tumor/Pathological Angiogenesis; Altered interstitial flow; VEGF presence	Mechanism study (Directional cues); Therapeutic target identification (alpha\beta integrin); Angiogenesis modulation	[32]
RNA sequencing analysis of early-stage atherosclerosis in vascular-on-a-chip; Collagen I (ECM gel); Mimetas (2-lane OrganoPlate)	HCAECs (Primary human coronary artery endothelial cells); Macrophages (derived from THP-1 monocytes)	Bidirectional flow; Rocker platform (7° angle, 8 min interval); Indirect exposure (via Macrophages); Pseudo-co-culture; Atherosclerosis Signaling pathway analysis; RNA-seq transcriptomics	Early-stage Atherosclerosis; Cigarette Smoke (CS) extract / HTP aerosol (via M0/M1 macrophages); Upregulated Atherosclerosis Signaling (in M1 indirect); Cell	Toxicology (Combustible cigarettes vs Heated tobacco products); Disease modeling (Endothelial-immune communication)	[33]

			death pathways (in Direct/M0 indirect)	; Risk assessment	
patient-derived isogenic iPSCs in 3D vessels on chip; Mimetas OrganoPlate	HHT1-hiPSC-ECs (Patient-derived); HBVPs (Primary Human Brain Vascular Pericytes)	Gravity-driven flow; Perfusion demonstrated by fluorescent beads & dextran; 3D Vascular networks; Isogenic control; EC-Pericyte interaction; Perfusion/Leakage assay	Hereditary Hemorrhagic Telangiectasia type 1 (HHT1); ENG gene haploinsufficiency; Defective vascular organization; Reduced pericyte coverage; Increased leakage; Reduced flow	Disease modeling (HHT1 pathophysiology); Mechanism study (EC-pericyte interaction defects); Drug discovery platform (Potential)	[34]
Vessel-on-a-Chip as a Viral Infection Model; PDMS (Polydimethylsiloxane); Glass (Cover); PDA (Polydopamine coating); Soft lithography fabricated	HUVECs (Human Umbilical Vein Endothelial Cells); Fibrin gel (Fibrinogen 4 mg/mL + Thrombin 4 U/mL)	Static culture with daily media change; Two parallel media channels design; 3D Vascular Network; Viral Mimicry; Inflammation Model; High-throughput screening potential	Viral Infection / Inflammation; Poly(I:C) (5 μg/mL, viral mimic); ↑VCAM-1, ↑ICAM-1, ↓VE-Cadherin integrity, ↓F-actin organization	Antiviral Drug Screening (RVX-208, JQ-1, PFI-1); Anti-inflammatory efficacy evaluation; Vascular pathophysiology study	[35]
Pro-metastatic cancer cells for in vitro extravasation models; PDMS (Chip, 3D printed mold) Hydrogel (Collagen I)	SKOV3 (Human ovarian cancer, 3D-sourced) or 2D-sourced cells; HUVEC (Human umbilical vein endothelial cells); NHLF (Normal human lung fibroblasts) (optional)	(Microfluidic, method not specified, but has endothelial channel); 3D co-culture; “Open-top” design; Models extravasation; 3D tumor source (hydrophobic surface-induced)	Cancer Metastasis/Extravasation (Induced by EGF or NHLF)	Drug screening (CCL2 inhibitor); Disease modeling; Pathogenesis study (importance of cell source)	[36]

vascularized micro-organ and micro-tumor models; DMS (Chip); (Membrane/Matrix not mentioned); Hughes lab (UCI) / Aracari Biosciences	Endothelial cells (EC) (Source not specified, but importance of source is stressed); Colon cancer cells (Cell line not specified)	Microfluidic, "perfused microvessels"); 3D vascularized; High-throughput (96-well format); Perfusable	Cancer (Colon cancer) / Angiogenesis (Induced by tumor cell co-culture)	Drug safety and efficacy evaluation (Fluorouracil, Vincristine, Sorafenib); Technology transfer validation	[37]
3D microengineered vascularized tumor spheroids;PDMS (Chip); Hydrogel (Fibrin);Soft lithography	HepG2 (Human hepatocellular carcinoma); HUVEC (Human umbilical vein endothelial cells)； HUVEC (Endothelial); LEC (Lymphatic endothelial cells); LF (Human lung fibroblasts)	Perfusion; tumor spheroid (hybrid); 3D vascular/lymphatic network (self-assembled); Angiogenesis/Lymphangiogenesis; Interstitial flow	Hepatocellular carcinoma; (Mimics) Tumor vascularization and metastasis	Drug screening (Axitinib); Disease modeling (TME); Pathogenesis study (angiogenesis/lymphangiogenesis)	[38]
Inflammation on a Progeria-on-a-Chip Model	HGPS iPS-SMCs (Smooth muscle cells from HGPS patient iPSCs); Healthy donor iPS-SMCs; (Also used primary SMCs)	Applies mechanical strain; Biomechanical strain (physiological/pathological)	HGPS (Progeria); Hypertensive phenotype; Inflammation; DNA damage (Induced by genetics (HGPS cells) + mechanical strain)	Disease modeling (vascular aging); Drug discovery; Pathomechanism study (biomechanics/inflammation)	[39]
Vein-Chip Recreates Venous Valve Architecture	Vascular endothelium； Fibrin； Platelets	Disturbed flow (simulated at valve cusps)； Fully vascularized venous valves; Virchow's triad simulation (Endothelium,	Deep Vein Thrombosis (DVT)； Induction: Cytokine stimulation； Traits: Prothrombotic	Preclinical approach; Venous pathophysiology; Antithrombotic drug treatment	[40]

		Flow, Blood constituents); Spatial adaptation	switch; Disturbed flow contribution		
Stem cell-derived vessels-on-chip; 3D-printed resin (body); Nylon filament (molding); Hydrogel (Collagen I)	hiPSC-derived Endothelial Cells (SC-ECs)	Gravity-driven; Rocking shaker; Open microfluidics; In-gel channel (via nylon filament removal); 3D sprouting angiogenesis	Atherosclerosis (induced by oxLDL and FFAs)	Disease modeling (vascular dysfunction); Pathomechanism study (scRNA-seq, proteomics); Animal-free research	[41]
Early calcific aortic valve disease by valve on chip; PDMS (Chamber); Elastic membrane; Hydrogel (Collagen-GAG)	pVIC (Porcine aortic valve interstitial cells); pVEC (Porcine aortic valve endothelial cells)	Uniaxial stretcher (Applies 10% cyclic strain); 3D Bilayered hydrogel (mimics fibrosa/spongiosa); Co-culture; Dynamic mechanical stimulation (Cyclic strain)	Calcific Aortic Valve Disease (CAVD) (Induced by diseased ECM + osteogenic media + mechanical strain)	Disease modeling (early CAVD); Pathogenesis study (proteomics/metabolomics)	[42]
Sensor-compatible vascular microphysiological system for endothelial injury; PDMS (Microchannels); Polyurethane (PU) membrane (anisotropic pores); Collagen I (Coating); Polyimide (Sensor substrate)	GFP-HUVECs (Green fluorescent protein-expressing Human umbilical vein endothelial cells)	Rocker (30° tilt every 25s); Bottomless stacked architecture (for sensor integration); Sensor-compatible design; Open-bottom structure; PU membrane support; Metabolic monitoring (Glucose/Lactate)	Drug-induced Vascular Injury; Minoxidil / Hydralazine; Disrupted VE-cadherin (Hydralazine); Decreased glycolytic flux (Hydralazine)	Drug toxicity screening; Metabolic analysis; Vascular disease modeling	[43]
Vascular Disease Modeling; Collagen I (3D-collagen gel)	mESCs (Mouse Embryonic Stem Cells, 129/Ola lines: Z/Red, R1, E14);	Static culture; 3D Sprouting Angiogenesis; Embryoid Bodies (EBs); Tip cell selection; Tube formation	Vascular Disease Modeling; Genetic deletions (e.g. embryonically lethal genes);	Drug testing (Phenotypic drug discovery); Gene function study; Cross-species comparison	[44]

		Endothelial cells (PECAM-1+)		Modulation of angiogenesis	(Mouse vs Human)	
	Lineage-specific vascular smooth muscle cell-on-a-chip; PDMS (Flexible membrane)	hiPSC-derived lineage-specific SMCs (Smooth muscle cells); (LM-SMCs, NC-SMCs, PM-SMCs); (Also used primary p-HASMCs)	Liquid pump (for media circulation); Vacuum pump (for mechanical stretch); Mechanical stretch (Tensile stress, 7.15% or 17.25%); Dynamic perfusion (recirculating); Lineage-specific cells	Aortic Aneurysm (AA); (Mimics) Fluoroquinolone drug response	Disease modeling; Pathogenesis study (segmental heterogeneity); Drug testing (Ciprofloxacin)	[45]
	Aorta smooth muscle-on-a-chip for thoracic aortic aneurysm; DMS (4 layers, incl. elastic film)	p-HASMCs (Primary human aortic smooth muscle cells) (from healthy donors or TAA patients); CRL1999 (cell line)	Vacuum pump (for mechanical stretch); Peristaltic pump (for drug delivery) High-throughput (18 wells); Mechanical stretch (20% strain); Multi-indicator detection (mitochondrial function)	Thoracic Aortic Aneurysm (TAA) (Modeled using patient-derived cells + mechanical strain)	Preclinical drug screening (HIF-1α inhibitors); Pathogenesis study (HIF-1α)	[46]
	Arteriole-on-a-chip for disease modeling	HUAECs (Human umbilical artery endothelial cells); HUSMCs (Human umbilical artery smooth muscle cells)	1 Hz oscillating pressure; Cyclic shear flow (stimulating arteriogenesis); Self-assembled arterioles; Vasculogenesis & Angiogenesis; SMC wrapping; Basement membrane; Vessel diameter adaptation (Primary enlargement/Secondary regression)	Arterial thrombosis (Induced by PMA)	Functionality testing (Dopamine induced vasodilation/vasoconstriction); Disease modeling (Thrombosis)	[47]
Blood	Bone Marrow cancer modle; PDMS; Standard soft lithography	EC (Endothelial Cells, cord blood-derived);	Hydrostatic pressure (driven by fluid level difference); Dual hexagonal	Cancer (MDA-MB-231) migration; Drug response	Hematopoiesis study; Leukocyte egress study; Drug response	[48]

	BMSC (Bone Marrow Stromal Cells); CD34+ HSPCs (Hematopoieti c Stem/Progenit or Cells) EC; hFOB 1.19 (Osteoblast cell line); CD34+ HSPCs	chambers (mimics dual niches); Perfusable vascular networks; Cell/molecule migration; 3D co-culture	(Doxorubicin, G-CSF)	testing; Cancer-bone marrow interaction	
Enhancement of Tumor Intravasation by organ on chip; Hydrogel (Collagen I); lrBM (Laminin-rich basement membrane)	MDA-MB-231 (Human breast cancer, RFP-labeled); TeloHAEC (Human aortic endothelial cells, GFP-labeled)	3D co-culture (tumor+vessel); Perfused microvessel; Mimics tumor-vascular interface	Cancer Metastasis/Intra vasation; Diabetes/Aging-related Dicarbonyl stress (Induced by Methylglyoxal (MG))	Disease modeling (cancer metastasis); Pathogenesis study (effect of dicarbonyl stress on intravasation)	[49]
3D Human Tumor Tissues Cultured in Dynamic Conditions; Alginate hydrogel (tunable stiffness); MIVO®; React4life	Single cell suspension Clonal spheroids; Circulating tumor cells (CTCs)	Fluid dynamic culture (mimicking physiological context); 3D embedding; Matrix stiffness tuning; Cell migration/spreadin g; Secondary site infiltration	Tumor growth & Metastasis; Clonal spheroid formation, CTC generation	Alternative in vitro disease models; Pre-clinical drug screening	[50]
Neutrophil phenotypes in sepsis patients；SynVivo Inc.	HLMVEC (Human lung microvascular endothelial cells);Neutrop hils (from patients or healthy donors)	flow rate is 1 μL/min；3D vascular network (topology); Tissue compartment; Porous (3 μm) interface; Shear stress	Sepsis (using patient neutrophils); Inflammation (Cytomix-induced)	Disease phenotyping (Hyperimmune, Hypoimmune, Hybrid); Immune cell functional analysis (adhesion/migra tion); Pathogenesis study	[51]
Lymphangion-chip with	LECs (Lymphatic	Microfluidic, "under flow");	Inflammation (Induced by	Preclinical research	[52]

Lymphatic Vessel	inflammation; GLP technique	endothelial cells); LMCs (Lymphatic muscle cells)	3D co-culture; Endothelial lumen; Circumferential muscle layers; Shear-induced cell alignment	pro-inflammatory cytokines); (Targets Lymphedema)	(mechanobiology, inflammation); Translational outcomes	
	Lymphatic Biology and Lymphatic Vessel/Tumor Coculture in a 3D Microfluidic Model; Collagen Type I (ECM gel, 4 mg/mL); Matrigel (ECM for organoids); MIMETAS BV (3-lane OrganoPlate, 40 chips/plate)	imLECs (Immortalized Human Lymphatic Endothelial Cells, hTERT/BMI-1 modified); Mouse Colon Cancer Organoids (mCRC-1/mCRC-2)	OrganoFlow rocker platform (±7° inclination, 8 min cycle, continuous perfusion); Long-lived imLECs (>12 months); Membrane-free; Lymphangiogenesis; Lumen formation; Barrier integrity (FITC-dextran assay)	Tumor-induced Lymphangiogenesis; Gradient of lymphangiogenic factors / Cancer organoids; Sprouting; Cancer cell motility/interaction	Lymphangiogenesis assay; Tumor-vessel interaction; Drug screening platform	[53]
Eye	Human Retinal Microvasculature-on-a-Chip; Hydrogel (Collagen I); Mimetas (OrganoPlate 2-lane)	hTERT hRMVECs (Immortalized human retinal microvascular endothelial cells); Also tested primary hRMVECs and HUVECs	Perfusion rocker (generates bidirectional flow); 3D tubular structure (not on membrane); Direct cell-ECM contact; Phase guide; Perfusion	Barrier leakage (induced by VEGFA or IL-1β)	Barrier biology study; Drug discovery; Drug (inhibitor) screening; Toxicity assessment	[54]
	Age-related macular degeneration by outer blood-retinal barrier; PDMS (Chip); Polyester membrane (8 μm); Hydrogel (Collagen I); CNC milled mold	HUVEC (Human umbilical vein endothelial cells); ARPE-19 (Human retinal pigment epithelial cell line); (Also used human lung fibroblasts)	Rocking platform (60° angle, 45s interval); Syringe pump (for permeability assay) 3D microvessel (formed by needle removal); 3D co-culture; Clinically relevant read-outs (OCT, Fluorescein angiography)	Age-related Macular Degeneration (AMD) (Induced by oxidative stress / H_2O_2)	Disease modeling; Drug screening; Pathogenesis study (barrier permeability)	[55]
Gum	Periodontal Disease	HGEC (Primary	Static perfusion (Natural diffusion,	Periodontal disease	Host-microbe interaction;	[56]

	Modeling Exploring Host-Microbe Interactions; PDMS (Chip); Glass (Substrate); Hydrogel (Collagen I + Matrigel + Fibronectin); Photolithography	human gingival epithelial cells); HGF (Primary human gingival fibroblasts); HUVEC (Human umbilical vein endothelial cells); THP-1 (Monocytes)	daily medium change) 4-channel (mimics sulcus/epithelium/connective tissue/vessel); 3D cell-hydrogel encapsulation; Immune cell perfusion	(Induced by Porphyromonas gingivalis (Pg))	Disease modeling; Drug discovery (Probiotic A. muciniphila)	
Lung	human lung airway-on-a-chip; PDMS; Emulate	Top: Primary Human Bronchial Epithelial (HBE) cells (Healthy or CF patient);Bottom: Primary Human Lung Microvascular Endothelial Cells (PMVEC)	Zoë® module (Automated perfusion, 30-45 μL/hr); Dual-channel; Porous membrane (7 μm); Air-Liquid Interface (ALI); Immune cell perfusion	Cystic Fibrosis (CF); Pseudomonas aeruginosa infection	Disease modeling; Pathophysiology study (inflammation/mucus/cilia); Host-pathogen interaction; Drug screening	[57]
	PLGA Nanofiber/PDMS Microporous Composite Membrane-Sandwiched Microchip; PDMS (Chip); PLGA nanofiber/PDMS microporous composite membrane; Soft lithography, Electrospinning	NCI-H1650 (Human lung adenocarcinoma); NCI-H460 (Human large cell lung cancer)	Syringe pump (1 mL/h); Composite membrane; Simulates alveoli shape (Membrane deformation); Low shear stress	Lung Cancer (Using NCI-H1650/NCI-H460 cell lines); Hypoxia (Induced by 1% O_2)	Drug evaluation (Gefitinib); Personalized treatment	[58]
	Lung microphysiological system in pathological and inflammatory reactions;	Top: BEAS-2B (Bronchial); NCI-H441 (Alveolar); THP-1 (Macrophages)	Peristaltic pumps (Takasago); (Nebulizer for transmission modeling);	Inflammation (Induced by LPS); COVID-19 (Induced by pseudovirus or spike protein)	Disease modeling (infectious disease); Cytokine storm study; Viral	[59]

PMMA (Chip body); PET (Porous membrane, 0.45 μm); PTFE (Membrane); CNC & adhesive	Bottom: HUVEC (Endothelial); THP-1 (Monocytes)	Dual-chamber (Bronchial/Alveolar); Air-Liquid Interface (ALI); Immune cell integration; Aerosol transmission system; Deep-learning analysis		transmission simulation	
A lung-on-chip model of early Mycobacterium tuberculosis infection; Emulate	Murine alveolar epithelial cells (ATs); Murine macrophages	Dual-channel; Air-Liquid Interface (ALI); Real-time time-lapse imaging	Tuberculosis (TB) (Induced by Mycobacterium tuberculosis (Mtb)); (Also models surfactant deficiency)	Disease modeling (early infection); Host-pathogen interaction; Pathogenesis study (role of pulmonary surfactant)	[60]
Impaired Wound Healing in a breathing lung on chip; PDMS (Sylgard 184); Ultra-thin elastic membrane (3.5 μm); Fibronectin (coating); AlveoliX AG (Related start-up)	A549 (Human lung alveolar epithelial-like cells); Fibroblasts (Mentioned in supplementary/discussion)	Static fluid (daily medium exchange); Electro-pneumatic system (for actuating microdiaphragm/breathing); "Breathing" motions; Cyclic mechanical stretch (10% linear strain, 0.2 Hz); Scratch assay (using micropipette tip); Ultra-thin membrane	Idiopathic pulmonary fibrosis (IPF) (relevant model); Impaired wound healing; Mechanical stress injury	Wound healing mechanism; Drug efficacy (rhHGF / Recombinant human hepatic growth factor)	[61]
Particulate Matter on Microfluidic Lung Chips; Glass (Chip body); Silicone elastomer (Channel); ITO (Indium tin oxide) (Electrodes); PET (Membrane);	Human Small Airway Epithelial Cells (SAEC); Human Pulmonary Alveolar Epithelial Cells (HPAEpiC)	Peristaltic pump (80 μl/ml); Integrated TEER sensor (ITO); Air-Liquid Interface (ALI); Dynamic perfusion (8 dyne/cm² shear stress); Real-time monitoring (microscope)	Inflammatory lung disease (COPD/Asthma) (Induced by PM10 and SLS / P.acnes)	Toxicology; Drug evaluation (Dexamethasone, Polyphyllin H); Disease modeling (PM10 exposure)	[62]

	BioSpero (Chip)					
	Lung-on-a-chip microdevice for anti-cancer drug testing; PDMS (Top layer); PLGA (Electrospun nanofiber membrane, ~3 μm thick)	A549 (Human non-small cell lung cancer); HFL1 (Human fetal lung fibroblasts); HUVEC (Human umbilical vein endothelial cells)	tatic culture; 3D cell culture (on nanofiber membrane); Mimics respiratory membrane; Co-culture	Lung Cancer (A549 cell line); (Mimics) Tumor invasion; (Mimics) Drug resistance	Drug evaluation (Gefitinib); Drug resistance mechanism study (HFL1/IGF-1); Tumor invasion modeling	[63]
	Immune cell in chip to model lung inflammation; PDMS (Chip); Glass (Substrate); Hydrogel (Fibrin)	HUVEC (Human umbilical vein endothelial) or hPMEC (Human pulmonary microvascular endothelial); 16HBE14o- (Human bronchial epithelial); PBMCs (Human peripheral blood mononuclear cells)	Peristaltic pump OR Rocking platform; 3-channel (epithelial/ECM/endothelial); Horizontal barrier; Live imaging; Tunable ECM stiffness	Lung inflammation (fMLP-induced chemotaxis); ARDS (Acute respiratory distress syndrome)	Immune cell migration/extravasation study; Inflammation mechanism study; Perfusion mode comparison (unidirectional vs. bidirectional)	[64]
	MSC-EVs to Acute Lung Injury Model on A Chip	Human alveolar epithelium; Human microvascular endothelium	Dynamic perfusion; Dual-channel (co-culture); Porous membrane; Air-liquid interface (ALI)	Acute Lung Injury (ALI) (Induced by LPS)	Drug screening (MSC-EVs); Pathophysiology study; Drug development	[65]
Esophagus	Patient-derived esophageal adenocarcinoma organ chip; PDMS; Emulate	Top: Patient-Derived Organoids (PDOs) (from EAC or adjacent normal tissue) Bottom: Patient-matched	Zoe-CM-1™ (Automated perfusion, 60 μl/h); Dual-channel (epithelial/stromal); Porous membrane (50 μm PDMS); Tumor-stroma interface; 3% O_2 (hypoxic) culture	Esophageal Adenocarcinoma (EAC) (patient-derived); (Mimics) Neoadjuvant chemotherapy (NACT) resistance	Functional precision oncology; Predicting chemotherapy response (NACT); Drug screening (Docetaxel-mbased)	[66]

		Fibroblasts (CAFs or normal fibroblasts)				
	Urban particulate matter-induced disruption of human respiratory mucosa using chip; PDMS (layers); PET membrane (0.4 μm pore, fibronectin coated); 3-layer stacked design	pHNE (Primary human nasal epithelial cells); WI-38 (Human lung fibroblast cell line); HUVEC (Human umbilical vein endothelial cells)	Static culture in device; Layer-by-layer assembly; 3D multicellular network; Triple-layered architecture; Epithelial-Endothelial crosstalk; Whole transcriptome analysis	Air pollution toxicity; Urban Particulate Matter (UPM) / TNF-α; Disrupted integrity (↓ZO-1, ↓Occludin, ↓VE-cadherin); Inflammation (↑ICAM-1, ↑IL-1β, ↑IL-6, ↑IL-8)	Toxicity screening (UPM effects); Disease modeling (Mucosal disruption mechanism); Gene expression profiling (RNA-seq)	[67]
	Primary Epithelial Airway Organ-on-Chip Platform for SARS-CoV-2 Therapeutic Screening; PREDICT96-ALI	Primary human bronchial / small airway epithelial cells (NHBEs) (from 3 donors)	Integrated micropumps (192 pneumatic pumps); High-throughput (96-well); Air-Liquid Interface (ALI); Perfusion (basal); BSL-3 compatible	SARS-CoV-2 infection (COVID-19) (Induced by virus (USA-WA1/2020))	Disease modeling; Viral replication kinetics study; Antiviral drug screening (Remdesivir, MPro61)	[68]
Gut	Cytokine induced inflammatory bowel disease model; Emulate	Top: Caco-2 (Human colorectal epithelium) Bottom: HUVEC (Human umbilical vein endothelium)	Dual-channel; Porous membrane; Mechanical stretch (mimics peristalsis, 2~10% at 0.15 Hz); Dynamic flow (30 μl/hr)	Inflammatory Bowel Disease (IBD) (induced by cytokines TNF-α and IFN-γ)	Disease modeling (IBD); Barrier integrity study; Cytokine signaling study	[69]
	Escherichia coli Heat-Stable Enterotoxin-Cyclic GMP in intestine chip; PDMS (Chip); Flexible permeable membrane;Emulate	Human jejunal enteroids (from adult stem cells)	(Microfluidic, method not specified); Mechanical stretch (Vacuum); Dual-channel; Air-Liquid Interface (ALI); Dynamic perfusion (Flow); Mechanical stretch	Enterotoxigenic E. coli (ETEC) infection (Induced by Heat-stable enterotoxin A (ST))	Host-pathogen interaction; Disease modeling (diarrhea); Pathogenesis study (cGMP signaling)	[70]

	Direct On-Chip Differentiation of Intestinal Tubules; Collagen I (ECM gel); Recombinant human Vitronectin (Coating); MIMETAS BV (OrganoPlate 3-lane)	hiPSCs (miFF1 line); Enterocytes (Villin+); Paneth cells (Lysozyme+); Neuroendocrine cells (CHGA+); Goblet cells (MUC2+); Stem cells (LGR5+)	OrganoFlow rocking platform (±7° inclination, 8 min interval); Directed differentiation (14 days); Tubular structure; Barrier function (TEER ~30 Ω cm², P-app); MMP-8 inhibition (prevents ECM invasion); Drug metabolism enzymes (CYP3A4, MDR1)	ntestinal Inflammation (IBD-like); Cytokine cocktail (TNF-α, IL-1β, IFN-γ); Gene upregulation (CCL20, IL-6, IL-8); Secretion (↑IL-6, ↑IL-8)	Disease modelling; Drug candidate screening (ADME/Tox); Permeability studies	[71]
	SARS-CoV-2 induced gut on chip; DMS (Chip); PDMS (Porous membrane, 5 μm); Soft lithography	Top: Caco-2 (Epithelial); HT-29 (Mucin-secreting) Bottom: HUVEC (Endothelial); (Also used PBMCs)	(Drive mechanism not specified, but flow rates: Top 200 μL/h, Bottom 50 μL/h) Dual-channel; Porous membrane; Epithelium-endothelium barrier; 3D Co-culture; Mucin secretion	COVID-19 (Intestinal infection) (Induced by SARS-CoV-2 virus (Strain 107) in the top channel)	Disease modeling; Virus-host interaction; Pathogenesis study (barrier injury, immune response); Drug development platform	[72]
	Gut on chip for inflammatory bowel disease; Glass (384-well plate based); Hydrogel (ECM); Mimetas (OrganoPlate 3-lane)	Caco-2 (Human colorectal epithelial cells)	Rocking platform (medium perfusion) 3D tubules; High-throughput (40 chips); PhaseGuide™; Membrane-free; Real-time TEER measurement	Inflammatory Bowel Disease (IBD) (Induced by cytokine cocktail (IL-1β, TNF-α, IFN-γ))	Disease modeling (IBD); Drug discovery (TPCA-1); Target validation (shRNA knockdown of RELA/MYD88)	[73]
Bone	Bone on chip for Osteoarthritis (OA); Hydrogel (Agarose);chiron	Human or bovine primary chondrocytes	Pressurized pump (300 mbar, 1 Hz); Dynamic biomechanical stimulation; 3D cell encapsulation	the osteoarthritis (OA) phenotype is induced by applying “dynamic biomechanical stimulation： 300 mbar of	OA pathophysiology modeling; Cartilage biology study; Evaluation of experimental therapeutics	[74]

			pressure at a frequency of 1 Hz (to model a "walking pace"), applied for 1 hour every day for 5 days.		
Osteochondral micro-physiological system in arthritis; GelMA + MeHA; Gelatin + Nano-HAp + Genipin; Fibrin/GelMA;3 D print	BM-hMSCs (Bone marrow derived human MSCs); BM-hMSCs + HUVECs	Biphasic bioreactor; Dual perfusion (Chondrogenic/Osteogenic); Continuous withdrawal (1.2 μL/min); Cartilage-Bone Crosstalk; Vascularized Bone; Biphasic media separation; Macrophage Conditioned Media (MCM)	Arthritis / Inflammation model; Cytokines (IL-1β, IL-6, TNF-α) / MCM; Catabolic response (↑MMPs, ↑ADAMTS); ECM degradation (↓COL2A1, ↓ACAN)	Disease modeling (Arthritis); Drug testing; Crosstalk study	[75]
Mimetic Biochip Model of osteoarthritis; ISSEEL fibrin hydrogel (animal-free); Biochip (In-house built); PMMA/PSA layers	Primary human fibroblast-like synoviocytes (FLS)(OA patients)	Feed microchannel connected to medium reservoirs; Diffusion-based; Animal-free; 3D organoids; Synovial architecture (Lining/Sublining structure); Condensation dynamics	Osteoarthritis (OA); TNF-α + IL-1β (Low dose pg/mL vs High dose ng/mL); Matrix softening; Synovial hyperplasia; Fibrosis (↑COL1A1, ↑COL3A1); Inflammation (↑IL6, ↑MMPs); Yap1 overexpression	Disease modeling (OA pathophysiology); Human-relevant research (Patient-specific); Drug screening platform	[76]
Chondrocyte culture-on-a-chip; PDMS (Bottom layer); Glass (Top layer)	Primary equine chondrocytes; Fibrin hydrogel	Diffusion-based nutrient gradient; Manual media exchange (providing periodic shear stress); Physiologic nutrient gradient;	Osteoarthritis (OA); Biochemical injury (TNF-α + IL-1β); ↑ADAMTS5, ↑IL-6, ↑MMPs (1, 3, 13), ↑ColX	Disease modeling (OA pathophysiology); Drug screening (Triamcinolone tested);	[77]

			Zonal organization (superficial/middle/ deep); Redifferentiation; 3D culture		Veterinary/Human modeling	
	Mechanically Active Osteoarthritis-on-Chip Model; PDMS (Flexible membrane) & Glass; BiomimX Srl (uBeat® technology)	Primary Human (hACs); Chondrocytes embedded in Fibrin gel (3D microtissues)	uBeat® MultiCompress Platform; Mechanical actuation; Confined Mechanical Compression (30% strain); Injectable channel; 3D Co-culture	Osteoarthritis (OA) / Inflammation; Induced by Hyperphysiological Compression (HPC) (30% strain, 1Hz)	Injectable Drug Screening (SYN321, Supartz)	[78]
	Monocyte extravasation of Intervertebral disc organ-on-a-chip; in house	NP cells (Nucleus Pulposus cells); THP-1 (monocyte-like cells)	Microfluidic chemotaxis chip; Models IVD geometry; Chemo-gradient channels; Immune cell infiltration	Nucleus Pulposus (NP) degeneration (induced by IL-1β)	Study monocyte extravasation, infiltration, and differentiation; Study immune response in degenerative IVD	[79]
Muscle	Muscular dystrophies in 3D-tissue-engineered-skeletal muscle; PDMS (mold); Hydrogel (Fibrinogen + Matrigel); Ecoflex Replica mold	hiPSC-derived Myogenic Progenitor Cells (MPCs)	Shaking platform (65 rpm) (dynamic culture); 3D-tissue-engineered-skeletal muscle; Force measurement (pillar deflection); Electrical stimulation; Gene knockdown (shRNA)	Duchenne muscular dystrophy (DMD); Limb-girdle muscular dystrophy type 2A (LGMD2A) (Both induced by shRNA delivered in hydrogel)	Disease modeling Pathogenesis study (proteomics) Preclinical drug evaluation (Micro-dystrophin)	[80]
	Enthesitis on chip for acute and chronic inflammation; PDMS (Chip housing); Polycarbonate / PC (Porous membrane);	Human Mesenchymal Stromal Cells (hMSCs); Differentiated Tenocytes (Top chamber); Differentiated Fibro chondrocytes	Dual-chamber Microfluidic Chip; Continuous perfusion (4 µL/h); Bilayer Co-culture; Selective differentiation; Porous barrier interaction	Acute Enthesitis (Induced by IL-17, IL-23, TNF-α / 3 days); Chronic Enthesitis & Ectopic Bone Formation (Induced by IL-17, IL-23, TNF-α / 21 days)	Disease modeling; Drug screening (Celecoxib / CXB)	[81]

	Collagen Type I (Coating/Overcast); Soft lithography	(Bottom chamber)				
	3D myotube contraction monitoring chip to model muscular dystrophies; PDMS (chip with micropillars and ridges)	Primary human myoblasts; Immortalized myoblasts (from healthy or L-CMD patients)	static culture Miniaturized (10-1000x fewer cells); 3D Myotube; Force measurement (pillar deflection); LIMA (light-induced surface patterning); Hydrogel-free	LMNA-related Congenital Muscular Dystrophy (L-CMD) (Induced by patient-derived cells)	Disease modeling (muscular dystrophy); Drug screening; Personalized medicine; Contraction analysis	[82]
Skin	Inflammatory skin on an organ-chip system; PDMS (Chip); PET (Porous membrane, 4 μm); Hydrogel (Collagen I)	HaCaT (Human keratinocyte cell line) Dermal fibroblasts	Liquid syringe (1.0 μL/min); Air pump (1.0 μL/min) (for ALI) "Interface-controlled" (IC-SoC); Air-Liquid Interface (ALI); Dynamic perfusion (basal); 3D Co-culture	Inflammatory skin disease (e.g., Acne) (Induced by Propionibacterium acnes (P.acnes) + SLS)	Toxicology; Drug evaluation (Dexamethasone, Polyphyllin H); Disease modeling (bacterial infection)	[83]
Adipose	Isogenic White Adipose Tissue Microphysiological System；PDMS (Chip slabs); PET (Porous membrane); Hyaluronic acid (Hydrogel); Soft lithography	Human iPSC (WTC-11 line); Isogenic; iADIPOs (Adipocytes); iMACs (M1/M2 Macrophages)	3D Microphysiological System (MPS); Under-the-membrane diffusion; Features: Crown-like structures (CLS); Macrophage infiltration; 3D Co-culture; Real-time observation	Chronic inflammation & Insulin resistance (Induced by M1 iMACs); Hypertrophy (Induced by Palmitic acid / PA); Aging (Simulated by Long-term culture / 74 days)	Disease modeling (Obesity/T2DM); Drug evaluation (Metformin, Anti-TNFα/IL6 Antibodies)	[84]
Placenta	Healthy and diseased placental barrier on-a-chip models; 384-well plate-based); Hydrogel	BeWo b30 (Human choriocarcinoma cell line, differentiated into syncytium); HUVEC	Rocking platform (OrganoFlow); 3-lane (Maternal/ECM/Fetal); High-throughput (40 chips); 3D co-culture;	Preeclampsia (induced by hypoxia/ischemia)	Disease modeling (preeclampsia); Pathological mechanism study; Drug/target screening	[85]

(Collagen-I / Collagen-IV): Mimetas (OrganoPlate 3-lane 40)	(Human umbilical vein endothelial cells)	PhaseGuide™; Membrane-free interface; Syncytium differentiation			
3D-Printed Model of the Feto-Maternal Interface for preterm birth therapies; BioMed Clear Resin (Formlabs); Parylene-C (Coating); GelMA (Hydrogel scaffold, 5.5% w/v)	DECs (Decidual cells, immortalized); AECs (Amnion epithelial cells, immortalized)	Static culture (96-well plate compatible); Microchannel array (diffusion-based communication); 3D printed scaffold; High-throughput; Two-chamber coculture; Parylene-C coating	Preterm Birth (PTB) / Inflammation; LPS / Poly(I:C); Inflammatory cytokines release (↑IL-6, ↑IL-8)	Drug screening (JAK/MAPK inhibitors); Preterm birth therapy discovery; Cytokine profiling	[86]
Feto-maternal interface organ-on-chip, models pregnancy pathology; PDMS; Soft lithography	AEC (Amnion epithelial); AMC (Amnion mesenchymal); CTC (Chorion trophoblasts); HUVEC (Umbilical vein endothelial); CTB (Cytotrophoblast, BeWo); STB (Syncytiotrophoblast, BeWo); DEC (Decidual cells)	Hydrostatic pressure (driven by reservoir fill height); Dynamic perfusion; 7-chambers (7 cell types); Mimics dual FMis; 3D Co-culture (w/ collagen)	Preterm Birth / Inflammation (Induced by LPS on maternal side)	Preclinical drug trial (eIL-10); Disease modeling; Drug delivery (EVs)	[87]
Environmental toxin cadmium using an organ-on-chip (FMi-OOC) model	Primary Human (Immortalized via HPV16 E6E7); Maternal: Decidua cells (DECs)	Four-chamber Microfluidic System; 4-layer Co-culture; Intercellular interactions maintenance; Propagation analysis	Preterm birth (PTB) risk / Inflammation (Induced by Cadmium / Cd); Decidual cell death (Induced by Cadmium / Cd)	Environmental toxin screening; Mechanism study (Maternal vs Fetal origin)	[88]

		Fetal: Chorion, Amnion mesenchymal & epithelial cells				
	Oxidative stress-associated disease using feto-maternal interface organ-on-a-chip; PDMS	Primary cells: Amnion epithelial; Mesenchymal; Chorion; Decidua	4 interconnected compartments (mimics FMi multi-layer structure); Microchannel connections	Oxidative Stress (OS); Preterm Birth (PTB) (Induced by Cigarette Smoke Extract (CSE))	Disease modeling; OS propagation study; Inflammatory pathway study	[89]
	Ascending infection with a feto-maternal interface organ-on-chip; PDMS (Chip); Hydrogel (Primary collagen, Matrigel, Collagen IV)	Primary cells (All from FMi): Decidua cells (Maternal) Chorion cells (CMCs/CTs) Amnion Mesenchymal cells (AMCs) Amnion Epithelial cells (AECs)	Hydrostatic pressure (driven by reservoir fill height); 4 concentric circular chambers (mimics FMi multi-layer structure); Microchannel connections	Ascending infection; Preterm birth (PTB) (Induced by LPS on the maternal side)	Modeling infection propagation; Inflammatory response study; Obstetrics pathology research	[90]
Breast	breast cancer metastasis-on-chip; (384-well plate based); Hydrogel (Collagen I); Mimetas (OrganoPlate® 3-lane)	MDA-MB-231 (Human breast cancer, GFP-labeled) or MCF7; Normal adult dermal fibroblasts HUVEC (Human umbilical vein endothelial cells)	Rocker platform (Bidirectional pulsatile flow, 2.5 dyne/cm^2);3D Co-culture (tumor+stroma); Perfused microvessel; PhaseGuide™; High-throughput screening (HCS)	Triple-negative breast cancer (TNBC) metastasis (Induced by cell co-culture)	Study of tumor invasion and intravasation; High-throughput drug screening; Drug response analysis (Imatinib)	[91]
Uterus	pathological cellular remodeling of the cervix; Two co-culture chambers; Microchannel connections	Ectocervical cells; Endocervical cells	Mimics ecto/endocervical regions	Inflammation/Infection; Preterm birth (Induced by LPS and TNFα)	Disease modeling (cervical remodeling); Obstetrics/gynecology research; Pathology studies	[92]

	A Human Cervix Chip for BV; PDMS (Chip-S1); ECM Coating (Collagen I/IV, Fibronectin);Emulate	Primary human cervical epithelial cells (CE cells); Primary human cervical stromal cells (CFs)	oë™ Culture Module (Automated); Intermittent flow (Apical: 0-30 μL/h, Basal: 40 μL/h); Epithelial-stromal interface; Mucus accumulation; Hormone responsiveness; Microbiome co-culture; Longitudinal monitoring	Bacterial Vaginosis (BV); Dysbiotic microbiome (Gardnerella vaginalis, Atopobium vaginae)	Preclinical models; Disease mechanisms (BV); Therapeutics/Diagnostics development; Host-microbiome interactions	[93]
	Primary Human Endometriotic Cells Based on Micro-Encapsulating Microfluidic Chip; Hydrogel Microcapsules (CMC core / Alginate shell); Electrospray technology	Primary Human (Patient-derived) Ectopic Endometrial Stromal Cells (hESCs)	Branched concentration gradient generator; Dynamic flow. Micro-encapsulation, 3D Spheroids, High-throughput.	Endometriosis Heterogeneity; Mimics patient-specific drug sensitivity & recurrence.	Personalized Drug Screening (Dienogest/Dydrogesterone), Omics analysis (Transcriptome).	[94]
Multi mode	human kidney and liver organoid-based multi-organ-on-a-chip model; TissUse (HUMIMIC Chip2)	Human kidney tubuloids (from adult cortical tissue); Human liver organoids (from healthy graft biopsies); Human bone marrow MSCs	Pulsatile pressure/vacuum driven (500 mbar, 0.5 Hz); Multi-organ connection (MOC); Organoid-based; 96-well insert format; Recirculating system	Acute kidney injury (AKI) (H_2O_2 induced)	(MSC-sEV) therapeutic efficacy study; (sEV) biodistribution analysis; (sEV) off-target liver effect analysis	[95]
	Liver- and pancreas-on-chip coculture for metabolic syndrome; PDMS (Sylgard 184); Replica molding with SU-8 master	Hepatocyte-like cells (HLCs, from hiPSCs); Pancreatic-like tissues (PLTs, β-cell spheroids, from hiPSCs)	Continuous nutrients/waste exchange; Tissues connected via fluidic tubing or integrated device; Organ crosstalk; Metabolic synergy; Transcriptomic	Metabolic Syndrome related; ↑CYP3A4 activity (Liver); ↑Glycogen storage (Liver); ↑C-peptide secretion	Disease modeling (Metabolic syndrome/Diabetes); Drug screening; Organ interaction study	[96]

		characterization; Advanced differentiation	(Pancreas); Inflammation markers (TGFβ/SMAD pathway)		
Multi-Organ-on-Chip approach to cancer therapy; PDMS (Chip); PC (Porous membrane, 2 μm); Hydrogel (Peptide); LCS platform	HepG2 (Human hepatocellular carcinoma cell line); MCF-7 (Human breast cancer cell line); HaCaT (Human keratinocyte cell line)	yringe pump (1.5 μL/min); Hybrid 3D/2D co-culture; Multi-organ connection; Perfusion	Breast Cancer (Modeled by MCF-7); Drug side effects (Skin toxicity)	Drug metabolism/efficacy/toxicity evaluation (5-FU, Capecitabine); Organ interaction study	[97]
Adipose tissue and Immune system on chip; Silicon (Chip); Glass (Substrate); Lithography and DRIE	Human Preadipocytes (HPADs); U937 (Human monocytic cell line)	Syringe pumps (neMESYS) (8 nL/s); Compartmentalized; Silicon-based; Porous barriers; Perfusion; Co-culture	Type 2 Diabetes / Insulin resistance (Inflammation induced by LPA)	Disease modeling; Organ interaction; Immune-metabolic study (cytokine/glucose uptake)	[98]
Lymphangiogenesis Model in Tumor Microenvironment; PDMS (Sylgard 184); Collagen Type I (Hydrogel); Polydopamine (Coating)	HDLECs (Human dermal lymphatic endothelial cells); Cancer spheroids - MDA-MB-231 (Invasive); BT474 (Non-invasive); A549 (Low-invasive)	Hydraulic head difference (generating interstitial flow); Flow range: 0.49 to 0.09 μm/s; Synergic stimulation (VEGF-A/C + Interstitial flow); 3D Lymphangiogenesis; Button-like junctions; Lumen formation; CCL21-CCR7 axis	Tumor Metastasis / Lymphangiogenesis; VEGF-A/C gradient + Interstitial flow; Lymphatic sprouting; Cancer cell invasion; Upregulation of Dll4, VEGFR3, CCL21	Disease modeling (Tumor microenvironment); Drug screening (Anti-lymphangiogenesis); Mechanistic study	[99]
Neuro-immune osteoarthritic micro pathophysiological system; OA micropathophysiological system /	Primary Human Chondrocytes; Mouse DRG Sensory Neurons； Primary Human M1	Microfluidic channel with patterned hydrogel; Integrated LoC sensor (EliChip™); Neuro-immune interactions; Axonal outgrowth;	Osteoarthritis (OA); M1 Macrophage secretome; Cartilage breakdown (↑MMP13, ↑COL10A1),	Disease modeling (OA pathophysiology); Molecular signature monitoring; Therapeutics development	[100]

EliChip™;GelMA (5% w/v); PDMS	Macrophages (Secretome)	3D patterning; Biosensing (IL-6, NGF)	Phenotype loss (↓COL2A1, ↓SOX9), Pathological innervation		
Malaria-on-a-Chip Phenotypic Disease for liver/spleen/blood/endothelium; PDMS (Poly-dimethyl siloxane); Acrylic (Chip housing); Hydrogel (Spleen embedding); Collagen (Coating for hepatocytes/splenocytes)	Primary human hepatocytes; Primary human splenocytes; HUVECs (Human Umbilical Vein Endothelial Cells); Erythrocytes (RBCs) infected with P. falciparum	Gravity-driven, physiological flow; Recirculating; Serum-free medium; Organ-organ interaction; Intraerythrocytic life cycle; Sequestration (of iRBCs); Digital twins / PK/PD modeling	Malaria; Hyperparasitemia (>5%); Drug resistance modeling (W2 strain)	Drug efficacy (Chloroquine, Lumefantrine, Artesunate); Off-target toxicity; Immune response (Cytokines: IL-4, TNF-α, etc.); Preclinical insight	[101]
Neurovascular Unit Response to Inflammatory Bone Condition	Primary mouse osteoclasts; HUVEC (Human umbilical vein endothelial cells); Embryonic DRG explants	3-compartment chip (Nerve/Vessel/Bone); Microgrooves (Nerve-Vessel); Micropillars (Vessel-Bone); 3D Hydrogel (Fibrin/Collagen)	Inflammatory bone disease (e.g., Osteoarthritis) (Induced by IL-1β in the bone chamber)	Disease modeling (OA); Neuro-vascular-bone interaction study; Pain (neuronal growth) study; Drug delivery system screening (nanoparticles)	[102]
Lung-liver interaction in infection fluidic chip; Silicone (chambers and tubing); Quasi Vivo (Kirkstall, UK) (QV600/QV500 chambers)	Primary Human Bronchial (HBEC) or Alveolar (ATC) Epithelial Cells (on Transwell); Huh-7 (Human hepatocellular carcinoma cell	Peristaltic pump (500 µL/min) Two-organ connection; Circulatory system (Closed loop); Air-Liquid Interface (ALI) (for lung); Modular (Transwell insert)	Bacterial infection/inflammation (Induced by inactivated NTHi and PAO1)	Organ interaction (lung-liver) study; Inflammatory response (secretome) study; Liver transcriptome analysis	[103]

	line) (on coverslip)				
Human gut and blood vessel (heart) model; PDMS (Chip); Glass (Substrate); Hydrogel (Collagen I)	Caco-2 (Human colorectal epithelium);SV EC4-10 (Murine endothelial cell line); MOVAS (Murine aortic smooth muscle cells);RAW 264.7 (Murine macrophages)	Peristaltic pump; 5-channel (epithelial/gel/vascular/gel/intima); Pillar spacing; Perfusion	Inflammatory Bowel Disease (IBD); Leaky Gut (LPS-induced)	Gut-heart axis study; (LPS-induced) barrier disruption study; Monocyte transmigration study	[104]
intestine-liver-heart-lung cancer microphysiological system; PDMS (3 layers);in house	Intestine: FHs 74 Int<br>Liver: THLE-2<br>Heart: HL-1<br>Lung Cancer: A549<br>(All are cell line-derived 3D spheroids)	uses microvalve array for flow control; High-throughput (16 chambers); Pneumatic microvalves; Switchable flow paths (Dual mode: organ formation / blood flow); Microwell arrays (for 3D spheroids)	Lung Cancer (A549-derived)	Multi-dimensional drug screening; Parallel testing of drug efficacy (anti-cancer) and side effects (multi-organ toxicity)	[105]
Lung and intestine chip for modeling human infectious diseases; PDMS; in house	HPAEpiC (Human pulmonary alveolar epithelial); HULEC-5a (Pulmonary microvascular endothelial); PBMCs (Human peripheral blood mononuclear cells); Caco-2/HT-29 (Intestinal epithelial);	Dual-channel; Porous membrane; Air-Liquid Interface (ALI) (for lung); Epithelial-endothelial barrier; Immune cell perfusion	SARS-CoV-2 infection (COVID-19); Intrauterine bacterial infection (also mentioned)	Viral pathogenesis study (host-pathogen interaction); Immune response study; Drug screening (Remdesivir)	[106]

	HUVECs (Human umbilical vein endothelial); Immune cells	

1. Schutgens, F., et al., *Tubuloids derived from human adult kidney and urine for personalized disease modeling.* Nat Biotechnol, 2019. **37**(3): p. 303–313.
2. Specioso, G., et al., *Apical Medium Flow Influences the Morphology and Physiology of Human Proximal Tubular Cells in a Microphysiological System.* Bioengineering (Basel), 2022. **9**(10).
3. Mou, X., et al., *An ultrathin membrane mediates tissue-specific morphogenesis and barrier function in a human kidney chip.* Sci Adv, 2024. **10**(23): p. eadn2689.
4. Jang, K.J., et al., *Human kidney proximal tubule-on-a-chip for drug transport and nephrotoxicity assessment.* Integr Biol (Camb), 2013. **5**(9): p. 1119–29.
5. Naik, S., et al., *A 3D Renal Proximal Tubule on Chip Model Phenocopies Lowe Syndrome and Dent II Disease Tubulopathy.* Int J Mol Sci, 2021. **22**(10).
6. Roye, Y., et al., *A Personalized Glomerulus Chip Engineered from Stem Cell-Derived Epithelium and Vascular Endothelium.* Micromachines (Basel), 2021. **12**(8).
7. Hiratsuka, K., et al., *Organoid-on-a-chip model of human ARPKD reveals mechanosensing pathomechanisms for drug discovery.* Sci Adv, 2022. **8**(38): p. eabq0866.
8. Wang, J., et al., *A virus-induced kidney disease model based on organ-on-a-chip: Pathogenesis exploration of virus-related renal dysfunctions.* Biomaterials, 2019. **219**: p. 119367.
9. Wang, L., et al., *A disease model of diabetic nephropathy in a glomerulus-on-a-chip microdevice.* Lab Chip, 2017. **17**(10): p. 1749–1760.
10. Wiriyakulsit, N., et al., *A model of hepatic steatosis with declined viability and function in a liver-organ-on-a-chip.* Sci Rep, 2023. **13**(1): p. 17019.
11. Negi, V., et al., *Modeling mechanisms underlying differential inflammatory responses to COVID-19 in type 2 diabetes using a patient-derived microphysiological organ-on-a-chip system.* Lab Chip, 2023. **23**(20): p. 4514–4527.
12. Ewart, L., et al., *Performance assessment and economic analysis of a human Liver-Chip for predictive toxicology.* Commun Med (Lond), 2022. **2**(1): p. 154.
13. Elci, B.S., et al., *Bioengineered Tubular Biliary Organoids.* Adv Healthc Mater, 2024. **13**(8): p. e2302912.
14. Vatine, G.D., et al., *Human iPSC-Derived Blood-Brain Barrier Chips Enable Disease Modeling and Personalized Medicine Applications.* Cell Stem Cell, 2019. **24**(6): p. 995–1005.e6.
15. Wu, Y., et al., *Modeling Ischemia-Reperfusion Injury in Stroke Using the BBB Chip.* ACS Omega, 2025. **10**(39): p. 45680–45695.
16. Ozgun, A., et al., *Unraveling the assembloid: Real-time monitoring of dopaminergic neurites in an inter-organoid pathway connecting midbrain and striatal regions.* Mater Today Bio, 2024. **25**: p. 100992.
17. Crilly, S. and M. Lomora, *Human in vitro models of neurovasculature and the application to pre-clinical intracerebral haemorrhage research.* Bioact Mater, 2026. **56**: p. 294–315.
18. Arellano, A., et al., *Attenuation of blood-brain barrier dysfunction by functionalized gold nanoparticles against amyloid-beta peptide in an Alzheimer's disease-on-a-chip model.* Mater Today Bio, 2025. **35**: p. 102453.

19. Tunesi, M., et al., *A miniaturized hydrogel-based in vitro model for dynamic culturing of human cells overexpressing beta-amyloid precursor protein.* J Tissue Eng, 2020. **11**: p. 2041731420945633.
20. Liu, J., et al., *An ischemic stroke-on-a-chip model integrated with machine learning for screening of drug candidates.* Lab Chip, 2025.
21. Saglam-Metiner, P., et al., *Differentiation of Neurons, Astrocytes, Oligodendrocytes and Microglia From Human Induced Pluripotent Stem Cells to Form Neural Tissue-On-Chip: A Neuroinflammation Model to Evaluate the Therapeutic Potential of Extracellular Vesicles Derived from Mesenchymal Stem Cells.* Stem Cell Rev Rep, 2024. **20**(1): p. 413–436.
22. Zhang, T., et al., *Unveiling the Heart's Hidden Enemy: Dynamic Insights into Polystyrene Nanoplastic-Induced Cardiotoxicity Based on Cardiac Organoid-on-a-Chip.* ACS Nano, 2024. **18**(45): p. 31569–31585.
23. Caluori, G., et al., *Non-invasive electromechanical cell-based biosensors for improved investigation of 3D cardiac models.* Biosens Bioelectron, 2019. **124-125**: p. 129–135.
24. Bracco Gartner, T.C.L., et al., *Cyclic strain has antifibrotic effects on the human cardiac fibroblast transcriptome in a human cardiac fibrosis-on-a-chip platform.* J Mech Behav Biomed Mater, 2023. **144**: p. 105980.
25. Mastikhina, O., et al., *Human cardiac fibrosis-on-a-chip model recapitulates disease hallmarks and can serve as a platform for drug testing.* Biomaterials, 2020. **233**: p. 119741.
26. Ates, B., et al., *Hydrogel-Integrated Heart-on-a-Chip Platform for Assessment of Myocardial Ischemia Markers.* ACS Omega, 2024. **9**(41): p. 42103–42115.
27. Wu, Q., et al., *SARS-CoV-2 pathogenesis in an angiotensin II-induced heart-on-a-chip disease model and extracellular vesicle screening.* Proc Natl Acad Sci U S A, 2024. **121**(28): p. e2403581121.
28. Wang, E.Y., et al., *An organ-on-a-chip model for pre-clinical drug evaluation in progressive non-genetic cardiomyopathy.* J Mol Cell Cardiol, 2021. **160**: p. 97–110.
29. Abudupataer, M., et al., *Construction of a Human Aorta Smooth Muscle Cell Organ-On-A-Chip Model for Recapitulating Biomechanical Strain in the Aortic Wall.* J Vis Exp, 2022(185).
30. Chen, M.B., et al., *A 3D microfluidic platform incorporating methacrylated gelatin hydrogels to study physiological cardiovascular cell-cell interactions.* Lab Chip, 2013. **13**(13): p. 2591–8.
31. Dittfeld, C., et al., *Challenges of aortic valve tissue culture - maintenance of viability and extracellular matrix in the pulsatile dynamic microphysiological system.* J Biol Eng, 2023. **17**(1): p. 60.
32. Shirure, V.S., et al., *Low levels of physiological interstitial flow eliminate morphogen gradients and guide angiogenesis.* Angiogenesis, 2017. **20**(4): p. 493–504.
33. Ohashi, K., et al., *RNA sequencing analysis of early-stage atherosclerosis in vascular-on-a-chip and its application for comparing combustible cigarettes with heated tobacco products.* Curr Res Toxicol, 2024. **6**: p. 100163.
34. Orlova, V.V., et al., *Vascular defects associated with hereditary hemorrhagic telangiectasia revealed in patient-derived isogenic iPSCs in 3D vessels on chip.* Stem Cell Reports, 2022. **17**(7): p. 1536–1545.
35. Kwon, J., et al., *Development of a Vessel-on-a-Chip as a Viral Infection Model and Antiviral Drug Screening Platform with Viral Mimics.* ACS Biomater Sci Eng, 2025. **11**(7): p. 4381–4393.
36. Lee, M., et al., *Hydrophobic surface induced pro-metastatic cancer cells for in vitro extravasation models.* Bioact Mater, 2024. **34**: p. 401–413.
37. Liu, Y., et al., *Human in vitro vascularized micro-organ and micro-tumor models are reproducible organ-on-a-chip platforms for studies of anticancer drugs.* Toxicology, 2020. **445**: p. 152601.
38. Ahn, J., et al., *3D microengineered vascularized tumor spheroids for drug delivery and efficacy testing.* Acta Biomater, 2023. **165**: p. 153–167.
39. Ribas, J., et al., *Biomechanical Strain Exacerbates Inflammation on a Progeria-on-a-Chip Model.* Small, 2017. **13**(15).

40. Rajeeva Pandian, N.K., et al., *Microengineered Human Vein-Chip Recreates Venous Valve Architecture and Its Contribution to Thrombosis.* Small, 2020. **16**(49): p. e2003401.
41. Marder, M., et al., *Stem cell-derived vessels-on-chip for cardiovascular disease modeling.* Cell Rep, 2024. **43**(4): p. 114008.
42. Tandon, I., et al., *A three-dimensional valve-on-chip microphysiological system implicates cell cycle progression, cholesterol metabolism and protein homeostasis in early calcific aortic valve disease progression.* Acta Biomater, 2024. **186**: p. 167–184.
43. Fu, Y., et al., *Development of a sensor-compatible vascular microphysiological system for metabolic monitoring during drug-induced endothelial injury.* Anal Sci, 2025. **41**(10): p. 1617–1625.
44. Galaris, G., et al., *In vitro Three-Dimensional Sprouting Assay of Angiogenesis using Mouse Embryonic Stem Cells for Vascular Disease Modeling and Drug Testing.* J Vis Exp, 2021(171).
45. Liu, G., et al., *A hiPSC-derived lineage-specific vascular smooth muscle cell-on-a-chip identifies aortic heterogeneity across segments.* Lab Chip, 2023. **23**(7): p. 1835–1851.
46. Zhu, S., et al., *Construction of a high-throughput aorta smooth muscle-on-a-chip for thoracic aortic aneurysm drug screening.* Biosens Bioelectron, 2022. **218**: p. 114747.
47. Shivani, S., et al., *Self-assembled human arteriole-on-a-chip for arterial functionality testing and disease modeling.* Lab Chip, 2025. **25**(20): p. 5162–5179.
48. Glaser, D.E., et al., *Organ-on-a-chip model of vascularized human bone marrow niches.* Biomaterials, 2022. **280**: p. 121245.
49. Kumar, N., et al., *Demonstration of Enhancement of Tumor Intravasation by Dicarbonyl Stress Using a Microfluidic Organ-on-chip.* Small, 2025. **21**(6): p. e2405998.
50. Pelizzoni, G. and S. Scaglione, *3D Human Tumor Tissues Cultured in Dynamic Conditions as Alternative In Vitro Disease Models.* Methods Mol Biol, 2023. **2572**: p. 203–210.
51. Yang, Q., et al., *Distinct functional neutrophil phenotypes in sepsis patients correlate with disease severity.* Front Immunol, 2024. **15**: p. 1341752.
52. Selahi, A., et al., *Lymphangion-chip: a microphysiological system which supports co-culture and bidirectional signaling of lymphatic endothelial and muscle cells.* Lab Chip, 2021. **22**(1): p. 121–135.
53. Frenkel, N., et al., *Long-Lived Human Lymphatic Endothelial Cells to Study Lymphatic Biology and Lymphatic Vessel/Tumor Coculture in a 3D Microfluidic Model.* ACS Biomater Sci Eng, 2021. **7**(7): p. 3030–3042.
54. Ragelle, H., et al., *Human Retinal Microvasculature-on-a-Chip for Drug Discovery.* Adv Healthc Mater, 2020. **9**(21): p. e2001531.
55. Arık, Y.B., et al., *Microfluidic organ-on-a-chip model of the outer blood-retinal barrier with clinically relevant read-outs for tissue permeability and vascular structure.* Lab Chip, 2021. **21**(2): p. 272–283.
56. Hu, Q., et al., *Gum-on-a-Chip Exploring Host-Microbe Interactions: Periodontal Disease Modeling and Drug Discovery.* J Tissue Eng, 2025. **16**: p. 20417314251314356.
57. Plebani, R., et al., *Modeling pulmonary cystic fibrosis in a human lung airway-on-a-chip.* J Cyst Fibros, 2022. **21**(4): p. 606–615.
58. Li, W., et al., *PLGA Nanofiber/PDMS Microporous Composite Membrane-Sandwiched Microchip for Drug Testing.* Micromachines (Basel), 2020. **11**(12).
59. Chen, Z., et al., *A storm in a teacup -- A biomimetic lung microphysiological system in conjunction with a deep-learning algorithm to monitor lung pathological and inflammatory reactions.* Biosens Bioelectron, 2023. **219**: p. 114772.
60. Thacker, V.V., et al., *A lung-on-chip model of early Mycobacterium tuberculosis infection reveals an essential role for alveolar epithelial cells in controlling bacterial growth.* Elife, 2020. **9**.
61. Felder, M., et al., *Impaired Wound Healing of Alveolar Lung Epithelial Cells in a Breathing Lung-On-A-Chip.* Front Bioeng Biotechnol, 2019. **7**: p. 3.

62. Jabbar, F., Y.S. Kim, and S.H. Lee, *Biological Influence of Pulmonary Disease Conditions Induced by Particulate Matter on Microfluidic Lung Chips.* Biochip J, 2022. **16**(3): p. 305–316.
63. Yang, X., et al., *Nanofiber membrane supported lung-on-a-chip microdevice for anti-cancer drug testing.* Lab Chip, 2018. **18**(3): p. 486–495.
64. van Os, L., et al., *Immune cell extravasation in an organ-on-chip to model lung inflammation.* Eur J Pharm Sci, 2023. **187**: p. 106485.
65. Chen, W., et al., *Screening Therapeutic Effects of MSC-EVs to Acute Lung Injury Model on A Chip.* Adv Healthc Mater, 2024. **13**(8): p. e2303123.
66. Pal, S., et al., *Patient-derived esophageal adenocarcinoma organ chip: a physiologically relevant platform for functional precision oncology.* J Transl Med, 2025. **23**(1): p. 577.
67. Byun, J., et al., *Identification of urban particulate matter-induced disruption of human respiratory mucosa integrity using whole transcriptome analysis and organ-on-a chip.* J Biol Eng, 2019. **13**: p. 88.
68. Fisher, C.R., et al., *A High-Throughput, High-Containment Human Primary Epithelial Airway Organ-on-Chip Platform for SARS-CoV-2 Therapeutic Screening.* Cells, 2023. **12**(22).
69. Tataru, C., et al., *Cytokine induced inflammatory bowel disease model using organ-on-a-chip technology.* PLoS One, 2023. **18**(12): p. e0289314.
70. Sunuwar, L., et al., *Mechanical Stimuli Affect Escherichia coli Heat-Stable Enterotoxin-Cyclic GMP Signaling in a Human Enteroid Intestine-Chip Model.* Infect Immun, 2020. **88**(3).
71. Naumovska, E., et al., *Direct On-Chip Differentiation of Intestinal Tubules from Induced Pluripotent Stem Cells.* Int J Mol Sci, 2020. **21**(14).
72. Guo, Y., et al., *SARS-CoV-2 induced intestinal responses with a biomimetic human gut-on-chip.* Sci Bull (Beijing), 2021. **66**(8): p. 783–793.
73. Beaurivage, C., et al., *Development of a Gut-On-A-Chip Model for High Throughput Disease Modeling and Drug Discovery.* Int J Mol Sci, 2019. **20**(22).
74. Peitso, V., et al., *Development of a Microphysiological Cartilage-on-Chip Platform for Dynamic Biomechanical Stimulation of Three-Dimensional Encapsulated Chondrocytes in Agarose Hydrogels.* Curr Protoc, 2024. **4**(12): p. e70079.
75. Smith, K.W.Y., et al., *Developing an in vitro osteochondral micro-physiological system for modeling cartilage-bone crosstalk in arthritis.* Front Immunol, 2025. **16**: p. 1495613.
76. Reihs, E.I., et al., *An Animal-Free Patient-Derived Tissue-Mimetic Biochip Model of the Human Synovial Membrane for Human-Relevant Osteoarthritis Research.* Adv Healthc Mater, 2025. **14**(23): p. e2404799.
77. Rosser, J., et al., *Microfluidic nutrient gradient-based three-dimensional chondrocyte culture-on-a-chip as an in vitro equine arthritis model.* Mater Today Bio, 2019. **4**: p. 100023.
78. Palma, C., et al., *An Advanced Mechanically Active Osteoarthritis-on-Chip Model to Test Injectable Therapeutic Formulations: The SYN321 Case Study.* Adv Healthc Mater, 2024. **13**(32): p. e2401187.
79. Son, H.G., et al., *Intervertebral disc organ-on-a-chip: an innovative model to study monocyte extravasation during nucleus pulposus degeneration.* Lab Chip, 2023. **23**(12): p. 2819–2828.
80. In 't Groen, S.L.M., et al., *A knock down strategy for rapid, generic, and versatile modelling of muscular dystrophies in 3D-tissue-engineered-skeletal muscle.* Skelet Muscle, 2024. **14**(1): p. 3.
81. Giacomini, F., et al., *Enthesitis on Chip - A Model for Studying Acute and Chronic Inflammation of the Enthesis and its Pharmacological Treatment.* Adv Healthc Mater, 2024. **13**(31): p. e2401815.
82. Rose, N., et al., *Bioengineering a miniaturized in vitro 3D myotube contraction monitoring chip to model muscular dystrophies.* Biomaterials, 2023. **293**: p. 121935.
83. Quan, Q., et al., *Analysis of drug efficacy for inflammatory skin on an organ-chip system.* Front Bioeng Biotechnol, 2022. **10**: p. 939629.
84. Qi, L., et al., *Human iPSC-Derived Proinflammatory Macrophages cause Insulin Resistance in an Isogenic White Adipose Tissue Microphysiological System.* Small, 2023. **19**(34): p. e2203725.

85. Rabussier, G., et al., *Healthy and diseased placental barrier on-a-chip models suitable for standardized studies.* Acta Biomater, 2023. **164**: p. 363–376.
86. Cherukuri, R., et al., *High-Throughput 3D-Printed Model of the Feto-Maternal Interface for the Discovery and Development of Preterm Birth Therapies.* ACS Appl Mater Interfaces, 2024. **16**(32): p. 41892–41906.
87. Safarzadeh, M., et al., *A multi-organ, feto-maternal interface organ-on-chip, models pregnancy pathology and is a useful preclinical extracellular vesicle drug trial platform.* Extracell Vesicle, 2024. **3**.
88. Kim, S., et al., *Molecular mechanisms of environmental toxin cadmium at the feto-maternal interface investigated using an organ-on-chip (FMi-OOC) model.* J Hazard Mater, 2022. **422**: p. 126759.
89. Richardson, L.S., et al., *Development of oxidative stress-associated disease models using feto-maternal interface organ-on-a-chip.* Faseb j, 2023. **37**(7): p. e23000.
90. Richardson, L.S., et al., *Modeling ascending infection with a feto-maternal interface organ-on-chip.* Lab Chip, 2020. **20**(23): p. 4486–4501.
91. Ozer, L.Y., et al., *Development of a cancer metastasis-on-chip assay for high throughput drug screening.* Front Oncol, 2023. **13**: p. 1269376.
92. Tantengco, O.A.G., et al., *Organ-on-chip of the cervical epithelial layer: A platform to study normal and pathological cellular remodeling of the cervix.* Faseb j, 2021. **35**(4): p. e21463.
93. Izadifar, Z. and D.E. Ingber, *A Human Cervix Chip for Preclinical Studies of Female Reproductive Biology.* Bio Protoc, 2025. **15**(7): p. e5262.
94. Chen, Q., et al., *Drug Screening of Primary Human Endometriotic Cells Based on Micro-Encapsulating Microfluidic Chip.* Adv Sci (Weinh), 2025. **12**(20): p. e2504647.
95. Nguyen, V.V.T., et al., *A human kidney and liver organoid-based multi-organ-on-a-chip model to study the therapeutic effects and biodistribution of mesenchymal stromal cell-derived extracellular vesicles.* J Extracell Vesicles, 2022. **11**(11): p. e12280.
96. Essaouiba, A., et al., *Transcriptomic characterization of the synergy between human induced pluripotent stem cells-derived liver- and pancreas-on-chip coculture.* Mol Cell Endocrinol, 2025. **606**: p. 112582.
97. Romanczuk, P., et al., *Multi-Organ-on-Chip approach to study the impact of inter-organ communication on the efficacy and side effects of cancer therapy.* Chem Biol Interact, 2025. **413**: p. 111460.
98. Ramadan, Q., S.B.N. Gourikutty, and Q.X. Zhang, *OOCHIP: Compartmentalized Microfluidic Perfusion System with Porous Barriers for Enhanced Cell-Cell Crosstalk in Organ-on-a-Chip.* Micromachines (Basel), 2020. **11**(6).
99. Cho, Y., et al., *Three-Dimensional In Vitro Lymphangiogenesis Model in Tumor Microenvironment.* Front Bioeng Biotechnol, 2021. **9**: p. 697657.
100. Kahraman, E., et al., *Deciphering cartilage neuro-immune interactions and innervation profile through 3D engineered osteoarthritic micropathophysiological system.* Mater Today Bio, 2025. **31**: p. 101491.
101. Rupar, M.J., et al., *Translation of a Human-Based Malaria-on-a-Chip Phenotypic Disease Model for In Vivo Applications.* Adv Sci (Weinh), 2025. **12**(38): p. e05206.
102. Neto, E., et al., *Micropathological Chip Modeling the Neurovascular Unit Response to Inflammatory Bone Condition.* Adv Healthc Mater, 2022. **11**(11): p. e2102305.
103. Reinhold, S., et al., *Modeling of lung-liver interaction during infection in a human fluidic organ-on-a-chip.* Sci Rep, 2025. **15**(1): p. 35241.
104. Sajin, D., et al., *Multi-organ-on-a-Chip: The Gut and Inflammatory Diseases.* ACS Biomater Sci Eng, 2025. **11**(9): p. 5330–5342.
105. Zhu, Y., et al., *Dynamic microphysiological system chip platform for high-throughput, customizable, and multi-dimensional drug screening.* Bioact Mater, 2024. **39**: p. 59–73.
106. Wang, Y., P. Wang, and J. Qin, *Microfluidic Organs-on-a-Chip for Modeling Human Infectious Diseases.* Acc Chem Res, 2021. **54**(18): p. 3550–3562.
